# Antioxidant Effect of *Lactobacillus fermentum* CQPC04-Fermented Soy Milk on D-Galactose-Induced Oxidative Aging Mice

**DOI:** 10.3389/fnut.2021.727467

**Published:** 2021-08-27

**Authors:** Xianrong Zhou, Hang-hang Du, Meiqing Jiang, Chaolekang Zhou, Yuhan Deng, Xingyao Long, Xin Zhao

**Affiliations:** ^1^Chongqing Collaborative Innovation Center for Functional Food, Chongqing Engineering Research Center of Functional Food, Chongqing Engineering Laboratory for Research and Development of Functional Food, Chongqing University of Education, Chongqing, China; ^2^Department of Food and Nutrition, College of Medical and Life Sciences, Silla University, Busan, South Korea; ^3^Department of Plastic Surgery, Chongqing Huamei Plastic Surgery Hospital, Chongqing, China

**Keywords:** soy isoflavones, peptides, *Lactobacillus fermentum*, soy milk, D-galactose

## Abstract

The aim of this study is to evaluate the changes in soy isoflavones and peptides in soy milk after lactic acid bacterial fermentation, and explore the positive effects of fermented soy milk on an oxidative aging mouse model induced with D-galactose. We found that free soybean isoflavones and peptides increased after soy milk was fermented by *Lactobacillus fermentum* CQPC04. The *in vivo* results indicated that *L. fermentum* CQPC04-fermented soy milk enhanced the organ index of the liver and spleen, and improved the pathological morphology of the liver, spleen, and skin. *L. fermentum* CQPC04-fermented soy milk increased the enzymatic activity of glutathione peroxidase (GSH-Px), total superoxide dismutase (T-SOD), and catalase (CAT), increased glutathione (GSH), but decreased the levels of nitric oxide (NO) and malondialdehyde (MDA) in serum, liver, and brain tissues of oxidative aging mice. The above mentioned fermented soy milk also increased the levels of collagen I (Col I), hyaluronic acid (HA), and collagen III (Col III), and decreased the levels of advanced glycation End products (AGEs) and hydrogen peroxide (H_2_O_2_). The RT-qPCR results showed that *L. fermentum* CQPC04-fermented soy milk upregulated the mRNA expression of nuclear factor erythroid 2?related factor (*Nrf2*), heme oxygenase-1 (*HMOX1*), quinone oxido-reductase 1 (*Nqo1*), neuronal nitric oxide synthase (*NOS1*), endothelial nitric oxide synthase (*NOS3*), Cu/Zn–superoxide dismutase (*Cu/Zn-SOD*), Mn–superoxide dismutase (*Mn-SOD*), and *CAT*, but downregulated the expression of inducible nitric oxide synthase (*NOS2*) and glutamate cysteine ligase modifier subunit (*Gclm*) in liver and spleen tissues. Lastly, the fermented soy milk also increased the gene expression of *Cu/Zn-SOD, Mn-SOD, CAT, GSH-Px*, matrix metalloproteinases 1 (*TIMP1*), and matrix metalloproteinases 2 (*TIMP2*), and decreased the expression of matrix metalloproteinase 2 (*MMP2*) and matrix metalloproteinase 9 (*MMP9*) in skin tissue. In conclusion, *L. fermentum* CQPC04-fermented soy milk was able to satisfactorily delay oxidative aging effects, and its mechanism may be related to the increase in free soy isoflavones and peptides.

## Introduction

The United Nations (UN) predicted that there would be an average annual increase rate of 2.5% in the elderly population from 1990 to 2020, and over the same period, the proportion of the world's aging population has risen from 6.6% in 1995 to 9.3% in 2020 (1). Aging causes a decline in physical function, but also can induce a variety of diseases, such as diabetes, osteoarthritis, cataracts, and cancer (2). For many countries, the increasing aging population will strain the economy and health care, and decrease the amount of available people in the labor pool (3). Therefore, research on how to delay aging or how to maintain health while aging is of great significance because it can improve the quality of life of the elderly, and reduce the burden on countries and families.

Fermented soybean products confer numerous health benefits. When soy milk is fermented by lactic acid bacteria or yeast, there is an increase in nutrients with greater bioavailability for the human body (4). Soy isoflavones and peptides are two bioactive components in soy milk that exist in the form of combined soy isoflavones and protein, which is not conducive to intestinal digestion and absorption (5). After microbial fermentation, the flavonoids are transformed into the aglycone form that is more easily absorbed by the human body, and the protein is broken down into amino acids and bioactive peptides, which thereby improve the nutritional value of soy milk (6).

The microorganisms that ferment soy milk also produce antioxidants. Studies have shown that *Lactobacillus brevis, Pediococcus pentosaceus* MYU 759, and *L. plantarum* promote the conversion of isoflavones and protein convert into aglycones and peptides, respectively, which possess increased antioxidant abilities in fermented soy milk against radicals such as 2,2'-azino-bis(3-ethylbenzothiazoline-6-sulfonic acid) (ABTS), 2,2-diphenyl-1-picryl-hydrazyl-hydrate (DPPH), and hydroxyl (7, 8). The metabolites produced during the fermentation of lactic acid bacteria, such as vitamins, exopolysaccharides, bacteriostatin, and short-chain fatty acids, also act in an anti-oxidant, hypolipidemic, and antibacterial capacity (9).

*Lactobacillus fermentum* CQPC04 is a new strain that was isolated and purified from natural fermented pickles, and in our previous research, we found that this strain exhibited satisfactory *in vitro* resistance to 0.3% bile salt and pH 3.0 artificial gastric juice, and increased the enzyme activity of T-SOD and CAT in the serum of mice with colitis (10). Based on our early results, we decide to further explore the effect of *L. fermentum* CQPC04 on D-galactose-induced oxidant aging in mice.

A natural aging model and accelerated aging model can be created, of which the accelerated aging model is used more frequently because of its shorter duration of study, higher survival rate of the animals, and easy application (11). D-galactose, an aldohexose that exists in many foods, is widely used to induce the aging animal model. The induction mechanism is that the catalysis of galactose oxidase can convert high levels of D-galactose into aldose and hydroperoxide, which will further induce the generation of reactive oxygen species (ROS), and cause mitochondrial dysfunction, oxidative stress, apoptosis, and inflammation (12). Vitamin C is a highly effective antioxidant in the human body and participates in many physiological and biochemical reactions. It is usually selected as a positive control sample for antioxidant experiments (13, 14).

In this study, *L. fermentum* CQPC04 was used to ferment soy milk, and then, the amounts of soy isoflavones and peptides were measured, and a peptide profile was obtained. The *L. fermentum* CQPC04-fermented soy milk was also administered to mice that underwent D-galactose-induced oxidative aging, and the organ indexes, serum and tissues indicators, and gene expression were determined and pathological observation was performed to evaluate the anti-aging effect of *L. fermentum* CQPC04-fermented soy milk.

## Materials and Methods

### Microbial Strain

Lactobacillus fermentum CQPC04 was preserved in the China General Microbial Culture Collection Center (CGMCC, Beijing, China) under accession number CGMCC 14493, and this strain was originally isolated from pickles in Chongqing, China by our group.

### Experimental Animals

Fifty 7-week-old female ICR mice were purchased from Chongqing Byrness Weil Biotech Ltd. [Chongqing, China, SCXK (XIANG) 2019-0004]. The mice were maintained in a room at constant temperature and humidity (25 ± 2°C, 50 ± 5%) with 12 h of light and 12 h of darkness, and they were allowed to ingest standard mouse chow and water *ad libitum*. All of the experiments were was performed according to 2010/63/EU directive and national standard of the people's Republic of China (GB/T 35892-2018) laboratory animal-guidelines for ethical review of animal welfare and institutional rules considering animal experiments. At the same time, the study protocol was approved by the Ethics Committee of Chongqing Collaborative Innovation Center for Functional Food (201905001B, Chongqing, China).

### Preparation of Fermented Soy Milk

According to a previous method with modification (15), *L. fer mentum* CQPC04 with a viability count of 1.0 × 10^5^ CFU/mL was added to sterilized soy milk, and then put in a constant temperature incubator (STIK Instrument Equipment Co., Ltd, Shanghai, China) for fermentation, the fermentation condition is 37°C, 12 h. The final viability count in the fermented soy milk was 1.3 × 10^8^ CFU/mL.

### Determination of Changes in Soy Isoflavones in Fermented Soy Milk

The soy isoflavones in soy milk were analyzed by HPLC (Ultimate 3000; Thermo Fisher Scientific, Inc., Waltham, MA, USA). Based on a previously published method with slight adjustments (16), 15 mL of non-fermented soy milk and fermented soy milk were each transferred to an Erlenmeyer flask containing 35 mL of 80% methanol (volume fraction), and ultrasonic extraction was conducted at room temperature for 4 h. Then, the extractions were centrifuged at 5,000 r/min for 12 min. After centrifugation, the samples were concentrated by rotary evaporation (Zhengzhou Great Wall Scientific Industrial and Trade Co. Ltd, Zhengzhou, Henan), and the volume was adjusted to 10 mL in a volumetric flask with 10% methanol solution. Subsequently, 1 mL of the extract was uniformly sampled and centrifuged at 10,000 r/min for 10 min, and then was drawn through a 0.22-μm filter membrane for HPLC measurement. Each experiment was repeated 3 times. Column parameters: mobile phase A: 0.1% acetic acid solution; mobile phase B: 0.1% acetic acid acetonitrile solution; wavelength: 260 nm; flow rate: 1 mL/min; column temperature: 40°C; injection volume: 20 μL. In addition, under the same chromatographic conditions, six standard products (daidzin, glycitin, genistin, daidzein, glycitein, and genistein) were used to make a standard curve with the concentration of 0.04, 0.12, 0.24, 0.36, and 0.48 mg/mL using chromatography-grade methanol.

### Analysis of Protein Profiles and Peptide Content

According to a previously described protocol (17), the protein profile was evaluated by sodium dodecyl sulfate-polyacrylamide gel electrophoresis (SDS-PAGE). The content of peptide was determined following a Chinese national standard code (GB/T 22492-2008).

### Grouping and Treatment of Experimental Animal

After adaptive feeding for 1 week, fifty female Kunming mice were equally distributed into five groups, consisting of the (i) normal group, (ii) D-galactose group (D-gal), (iii) D-galactose + vitamin C group (D-gal+VC), (iv) D-galactose + *L. fermentum* CQPC04-fermented soy milk group (D-gal + CQPC04), and (v) D-galactose + non-fermented soy milk group (D-gal + NO). The groups and their specific treatments for the entire experimental period are presented in Supplementary Figure 1.

### Sample Collection and Preservation

All mice were fasted with water for 16 h and depilated on the back before being euthanized by severing the spinal cord. Whole blood was collected and centrifuged at 4,000 rpm for 10 min at 4°C to obtain the upper serum. Then, the liver, spleen, and whole brain were weighed to calculate the organ indexes. The organ indexes were determined by the following formula: C (%) = (m1/m2) × 100, in which C represents the organ index, m1 denotes the organ weight, and m2 denotes the body weight of the mice.

### Pathological Observation of Liver, Spleen, and Skin Tissues

Soybean-sized pieces of tissue of the liver, spleen, and skin were harvested and fixed in 10% paraformaldehyde solution for 48 h. Then all of the above tissues were delivered to Wuhan Sevicebio Biotechnology Co., Ltd. (Wuahn, China) for pathological detection. Lastly, an upright microscope (Olympus Scientific Instruments Co., Ltd., Tokyo, Japan) was used to observe the morphology of the tissues and obtain photographs.

### Measurement of Oxidative Indicators

The levels of GSH-Px, GSH, T-SOD, MDA, NO, and CAT in serum were directly determined using conventional biochemical kits and following the manufacturer's instructions (NanJing JianCheng Bioengineering Institute, Nanjing, China). In addition, it was necessary to pre-treat the mouse liver and brain tissues before measuring the above indicators, which consisted of homogenizing one gram of tissue and 9 mL of physiological saline with a tissue homogenizer (Hangzhou Aosheng Biotechnology Co., Ltd, Hangzhou, China), centrifuging the homogenate at 12,000 rpm for 10 min at 4°C, and collecting the supernatant as the measurement sample for the above indicators.

### Measurement of Hydrogen Peroxide (H_2_O_2_), Collagen (Col) I, Col III, Advanced Glycation End Products (AGEs), and Hyaluronic Acid (HA) Levels in Skin

The skin pretreatment method used was same as that described in Section Measurement of Oxidative Indicators. The amount of hydrogen peroxide in the skin was determined using conventional biochemical kit (NanJing JianCheng Bioengineering Institute, Nanjing, China), and Col I, AGEs, Col III, and hyaluronic acid levels were measured by the enzyme-linked immunosorbent assay (ELISA) kits (Shanghai Enzyme Biotechnology Co., Ltd., Shanghai, China).

### Quantitative Real-Time PCR (RT-qPCR) Assay

The total RNA in the liver, spleen, and skin of the mice was extracted using TRIzol reagent (Thermo Fisher Scientific, Inc., Waltham, MA, USA). Then, the mRNA was reverse transcribed into cDNA using a cDNA kit (Thermo Fisher Scientific, Waltham, MA, USA). Finally, RT-qPCR (reverse transcription-quantitative polymerase chain reaction) was performed, and a real-time fluorescence quantitative PCR instrument (Thermo Fisher Scientific, Waltham, MA, USA) was used to detect the mRNA expression of target genes. All primers were from Invitrogen (Thermo Fisher Scientific, Waltham, MA, USA), and their sequences are listed in Table 1. Finally, the relative expression of the target genes was calculated using the formula 2^−Δ*ΔCT*^, in which β-actin was chosen as the internal reference marker (18).

**Table 1:** Sequences of the primers.

**Gene**	**Sequences**	**Reference sequence**
*Nrf2*	F:5'- CTTTAGTCAGCGACAGAAGGAC−3'	NM_010902.4
	R:5'- AGGCATCTTGTTTGGGAATGTG−3'	
*HMOX1*	F:5'-AGGTACACATCCAAGCCGAGA-3'	NM_010442.2
	R:5'-CATCACCAGCTTAAAGCCTTCT-3'	
*Nqo1*	F:5'-AGGATGGGAGGTACTCGAATC-3'	NM_008706.5
	R:5'-TGCTAGAGATGACTCGGAAGG-3'	
*NOS1*	F:5'-TCCCAGTAACGGACCTCAG-3'	NM_008712.3
	R:5'-TGCTCAACACAGGTTCTATCTCT-3'	
*NOS2*	F:5'-GTTCTCAGCCCAACAATACAAGA-3'	NM_010927.4
	R:5'-GTGGACGGGTCGATGTCAC-3'	
*NOS3*	F:5'-TCAGCCATCACAGTGTTCCC-3'	NM_008713.4
	R:5'-ATAGCCCGCATAGCGTATCAG-3'	
*Cu/Zn-SOD*	F:5'-AACCAGTTGTGTTGTCAGGAC-3'	NM_011434.1
	R:5'-CCACCATGTTTCTTAGAGTGAGG-3'	
*Mn-SOD*	F:5'-CAGACCTGCCTTACGACTATGG-3'	NM_013671.3
	R:5'-CTCGGTGGCGTTGAGATTGTT-3'	
*GSH-Px*	F:5'-CCACCGTGTATGCCTTCTCC-3'	NM_008160.6
	R:5'-AGAGAGACGCGACATTCTCAAT-3'	
*Catalase*	F:5'-GGAGGCGGGAACCCAATAG-3'	NM_009804.2
	R:5'-GTGTGCCATCTCGTCAGTGAA-3'	
*Gclm*	F:5'-AGGAGCTTCGGGACTGTATCC-3'	NM_008129.4
	R:5'-GGAAACTCCCTGACTAAATCGG-3'	
*TIMP1*	F:5'-CGAGACCACCTTATACCAGCG-3'	NM_001044384.1
	R:5'-ATGACTGGGGTGTAGGCGTA-3'	
*TIMP2*	F:5'-TCAGAGCCAAAGCAGTGAGC-3'	NM_011594.3
	R:5'-GCCGTGTAGATAAACTCGATGTC-3'	
*MMP2*	F:5'-ACCTGAACACTTTCTATGGCTG-3'	NM_008610.3
	R:5'-CTTCCGCATGGTCTCGATG-3'	
*MMP9*	F:5'-GCAGAGGCATACTTGTACCG-3'	NM_013599.4
	R:5'-TGATGTTATGATGGTCCCACTTG-3'	
*β-actin*	F:5'-CATGTACGTTGCTATCCAGGC-3'	NC_000071.7
	R:5'-CTCCTTAATGTCACGCACGAT-3'	

### Statistical Analysis

Graph Pad Prism 7.0 and SPASS 17.0 software were used to calculate and analyze results, and the results presented as the mean ± standard deviation ($\bar{\text{X}}$ ± SD). Comparison of the differences between the datasets was conducted using analysis of variance Duncan's multiple range test. When *p* < 0.05 indicates statistical significance.

## Results

### Quantitation of Soy Isoflavones

**Figure 1:**
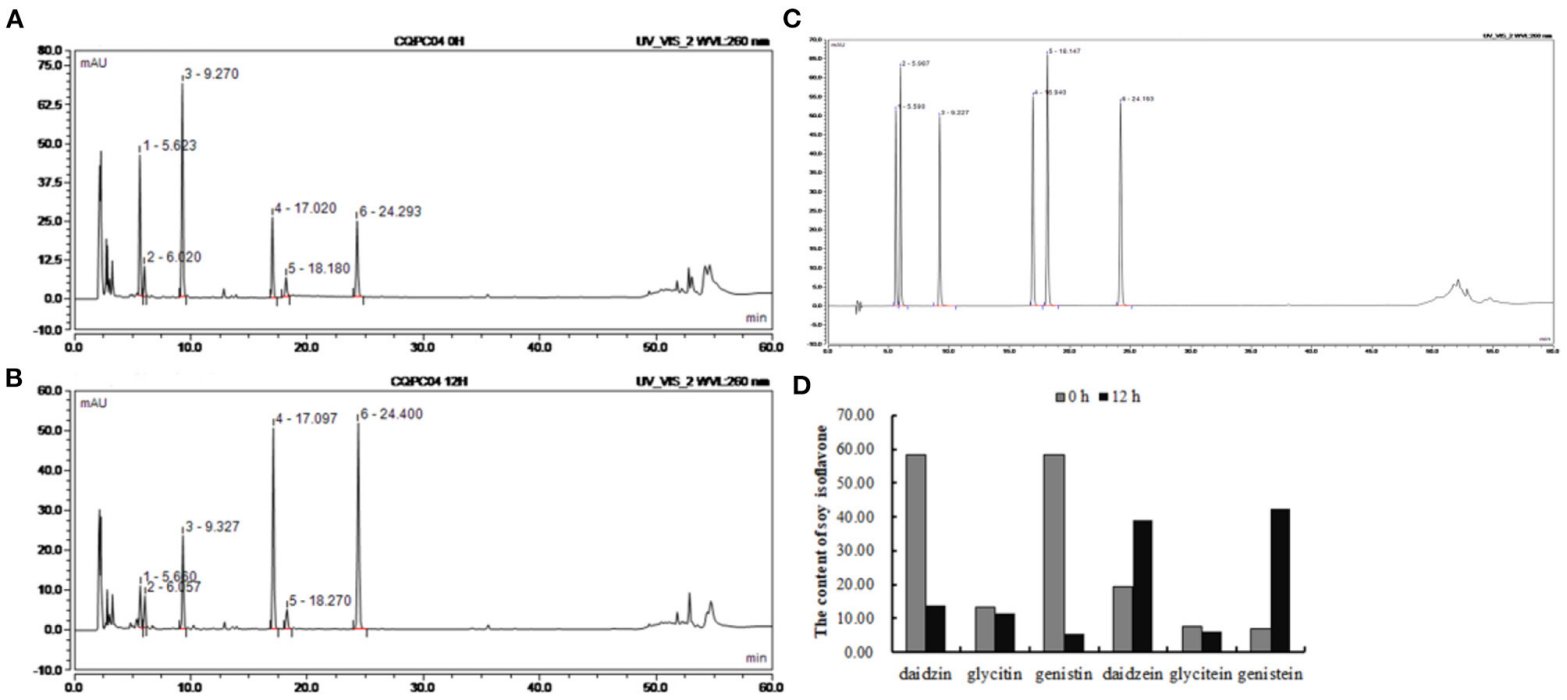
Changes of soy isoflavone in soy milk before and after fermentation. **(A)** liquid chromatogram of non-fermented soy milk; **(B)** liquid chromatogram of *L. fermentum* CQPC04-fermented soy milk; **(C)** liquid chromatogram of standards; **(D)** soy isoflavone contents in non-fermented soy milk and *L. fermentum* CQPC04-fermented soy milk. 1: daidzin; 2: glycitin; 3: genistin; 4: daidzein; A; 5: glycitein; and 6: genistein.

The contents of isoflavones in soy milk before and afther fermentation were determined by HPLC (Figures 1A–C). It can be seen from Figure 1D that daidzin, glycitin, genistin, daidzein, glycitein, and genistein were present in unfermented soy milk at 58.35, 13.17, 58.27, 19.41, 7.55, and 6.87 mg/mL. However, there was less daidzin, glycitin, and genistin in *L. fermentum* CQPC04-fermented soy milk at 13.78, 11.44, 5.34 mg/mL, whereas daidzein and genistein were increased to 39.06 and 42.39 mg/mL, respectively, and glycitein was decreased to 5.931 mg/mL. These results indicate that *L. fermentum* CQPC04 transformed conjugated soy isoflavones to free soy isoflavones, which are more easily absorbed by the body.

### Peptide Content and Protein Profiles

**Figure 2:**
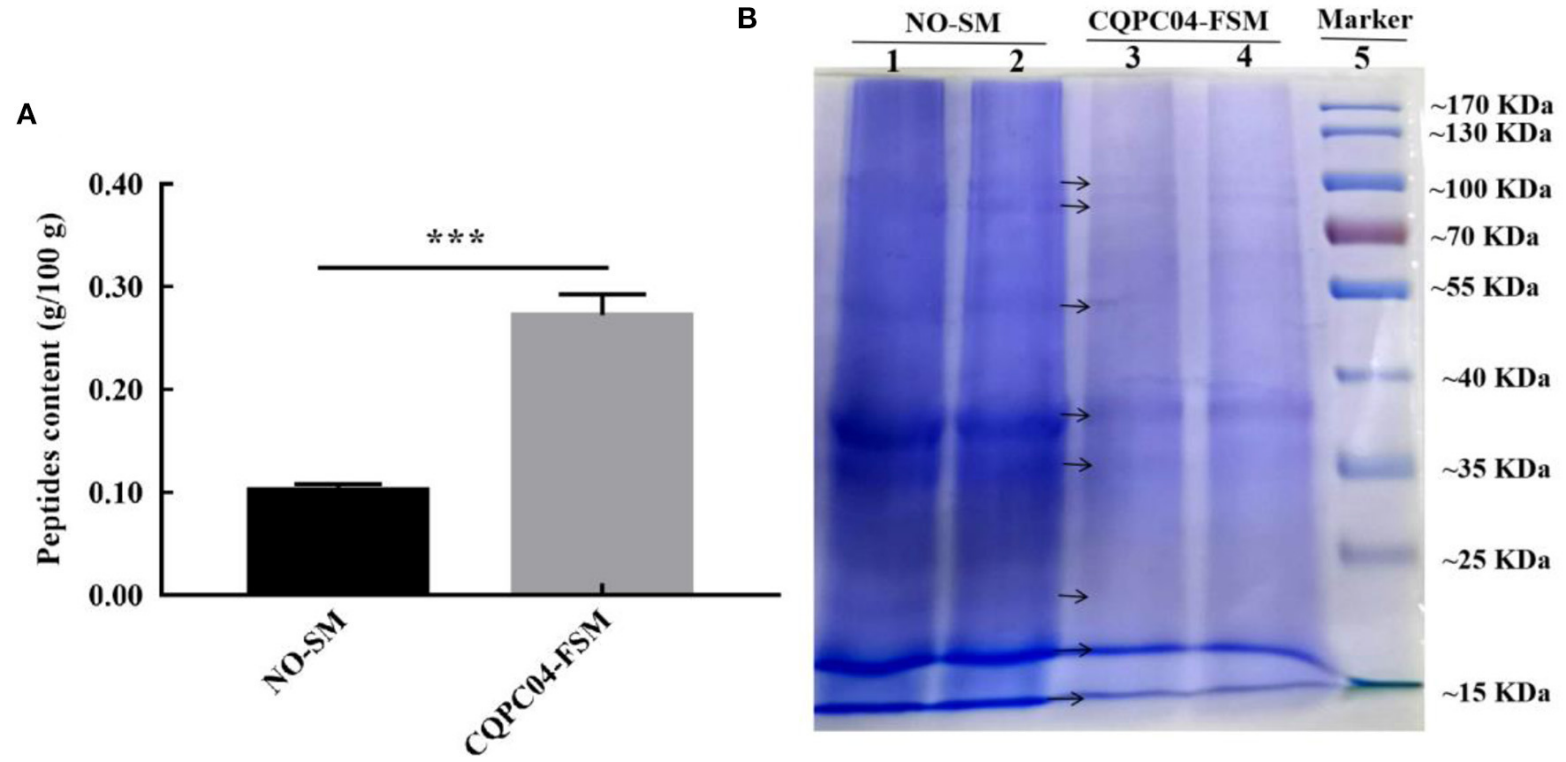
Peptide contents and protein profiles in soy milk before and after fermentation. **(A)** peptide contents in non-fermented and *L. fermentum* CQPC04-fermented soy milk; **(B)** protein profiles of non-fermented and *L. fermentum* CQPC04-fermented soy milk. NO-SM: non-fermented soy milk; CQPC04-FSM: *L. fermentum* CQPC04-fermented soy milk. ***p < 0.001 compared to D-gal group.

The peptide content and protein profiles are two useful indicators that can reflect the changes in the protein in soy milk before and after fermentation (Figure 2). From Figure 2A, we can see that the peptide content in non-fermented soy milk was 0.10 ± 0.006 g/100 g, while it significantly increased to 0.26 ± 0.015 g/100 g after the soy milk was fermented by *L. fermentum* CQPC04 (*p* < 0.05). Figure 2B shows that the macromolecular proteins in non-fermented soybean milk were significantly higher than those in soy milk fermented with *L. fermentum* CQPC04 (*p* < 0.05). The above results indicate that fermentation with *L. fermentum* CQPC04 can convert the macromolecular proteins in soy milk into small-molecule peptides.

### Organ Index of Liver and Spleen

**Figure 3:**
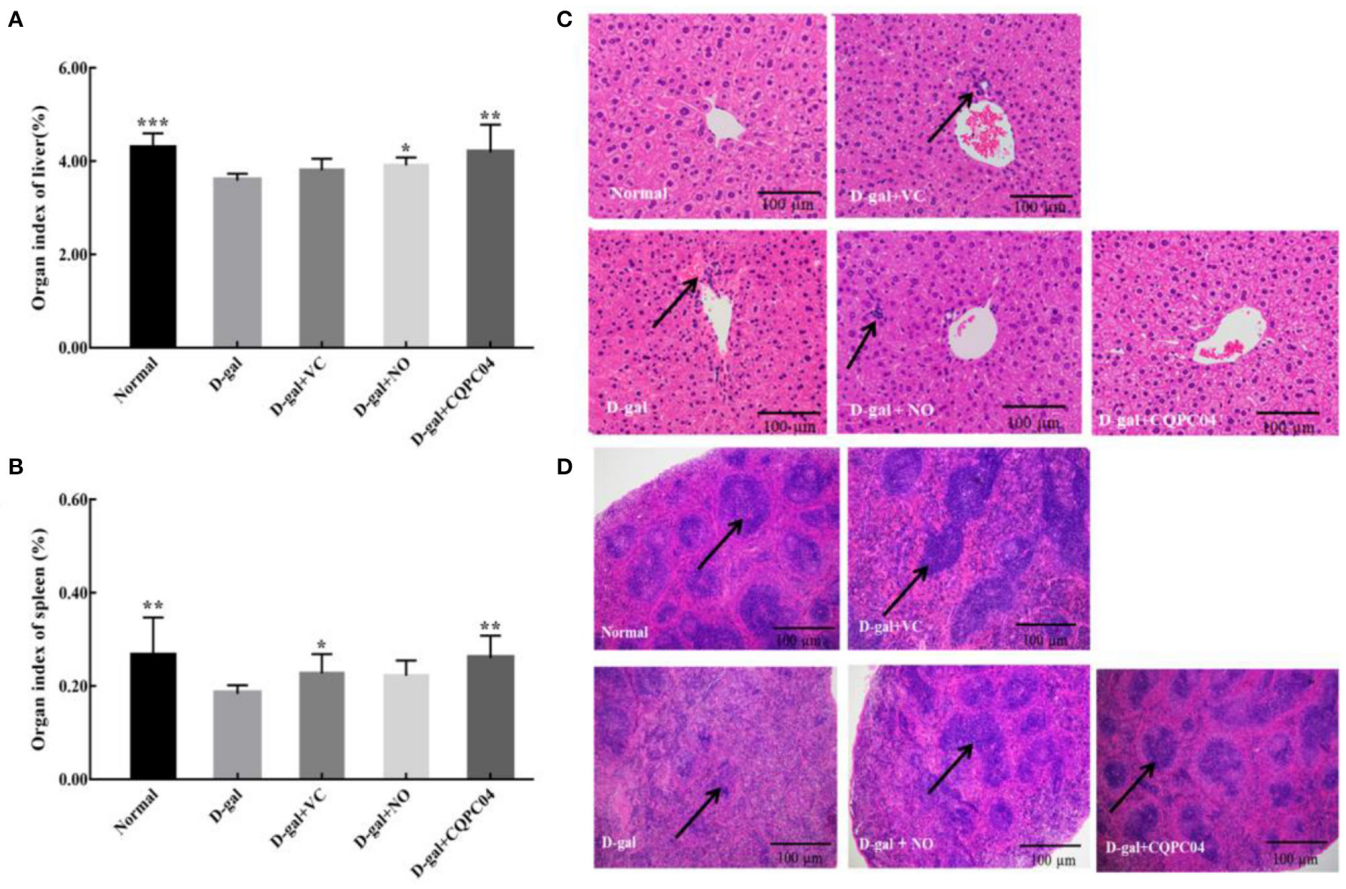
Organ index and pathological observation of liver and spleen. **(A)** organ index of liver; **(B)** organ index of spleen; **(C)** pathological observation of liver; **(D)** pathological observation of spleen. D-gal: mice fed the standard chow diet plus drinking water with intraperitoneal injection of D-galactose (120 mg/kg of BW); D-gal + VC: vitamin C (100 mg/kg of BW) plus intraperitoneal injection of D-galactose (120 mg/kg of BW); D-gal + NO: non-fermented soy milk (0.1 mL/10 g of BW) plus intraperitoneal injection of D-galactose (120 mg/kg of BW); D-gal + CQPC04: *L. fermentum* CQPC04-fermented soy milk (0.1 mL/10 g of BW) plus intraperitoneal injection of D-galactose (120 mg/kg of BW). *p < 0.05, **p < 0.01, and ***p < 0.001 compared to D-gal group. **(C)** the black arrows indicate necrotic cells, and the black bar represents 100 μm. **(D)** the black arrows indicate white pulp, the black bar represents 100 μm.

There is usually degeneration or atrophy of organs during aging. As shown in Figures 3A,B, the D-gal group possessed the lowest organ indexes of the liver and spleen, while they are highest in the normal group. Compared to the D-gal group, the organ indexes of the liver and spleen increased to varying degrees after the mice were treated with non-fermented soy milk, VC, and *L. fermentum* CQPC04-fermented soy milk, in which the organ indexes in the D-gal + CQPC04 group increased more than those in the other two groups and were similar to those of the normal group.

### Pathological Morphology of the Liver

Figure 3C indicates that the liver structure of healthy mice was complete, and the hepatic cells were scattered around the central vein in the form of satellite emission, with no necrotic cells or inflammatory cells. On the contrary, the liver cells of mice in the D-gal group were disorganized with a large number of necrotic cells. Although the liver cells in the D-gal + VC group and the D-gal + NO group were partially necrotic, the overall cellular shape was obviously more normal than that of the D-gal group. On the other hand, the liver structure of mice in the D-gal + CQPC04 group appeared similar to that of the normal group, with no necrotic or inflammatory cells.

### Pathological Observation of the Spleen

The histological features of the spleen sections stained with H&E are presented in Figure 3D. Compared with the normal group, the percentage of white pulp of the spleen in the D-gal group clearly decreased, and there was no clear boundary between the red pulp and white pulp. However, the VC, non-fermented soy milk, and *L. fermentum* CQPC04-fermented soy milk groups all displayed an increase in the percentage of white pulp to varying degrees. Moreover, the most white pulp was observed in the D-gal + CQPC04 group, which is similar to the normal group.

### Pathological Observation of the Skin

**Figure 4:**
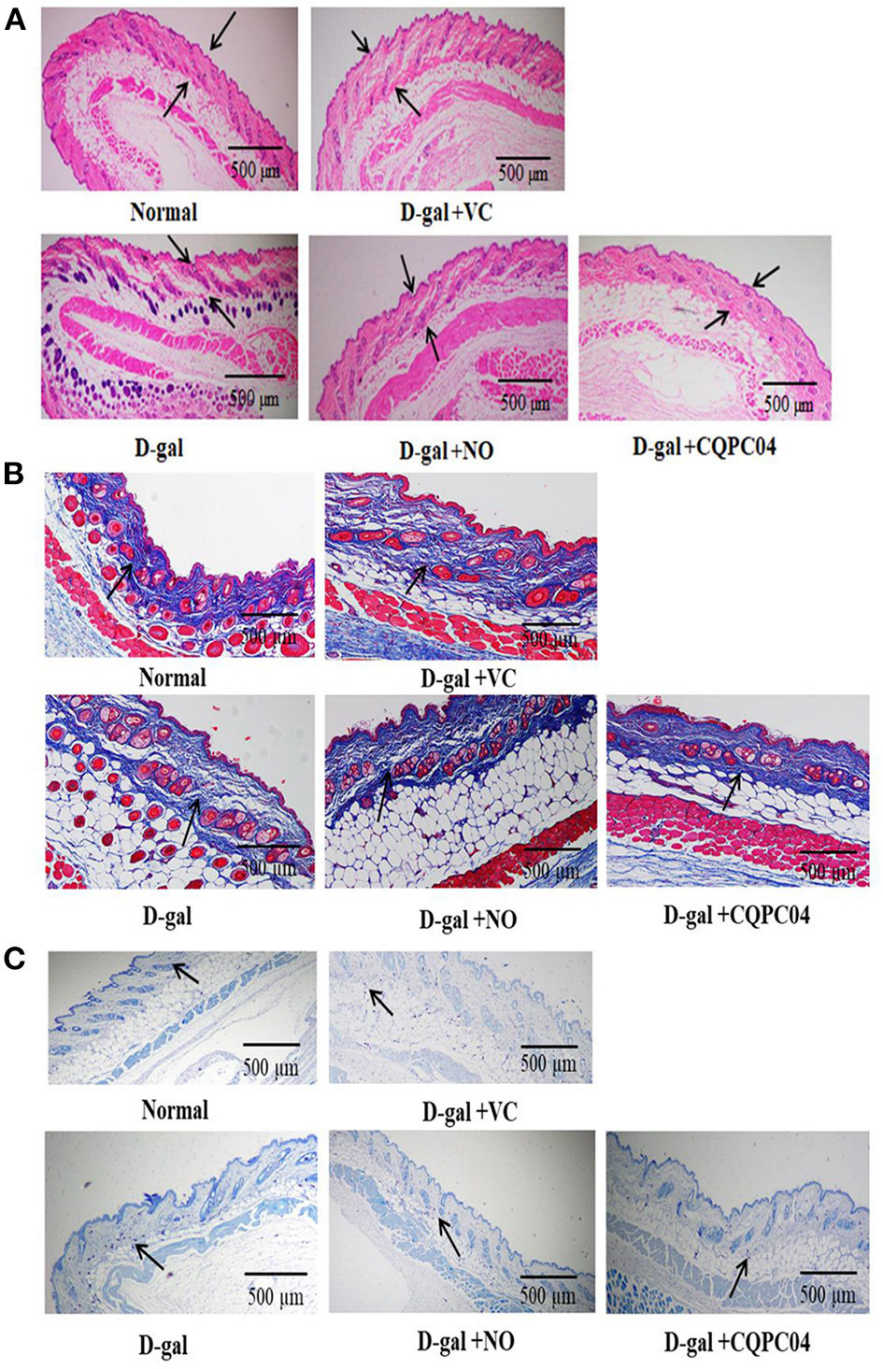
Pathological observation of skin. **(A)** H&E stain of skin, the black arrows indicate corium layer, and the black bar represents 500 μm; **(B)** Masson stain of skin, the black arrows indicate collagen fibers, and the black bar represents 500 μm; **(C)** Toluidine blue stain of skin, the black arrows indicate mast cells, and the black bar represents 500 μm. D-gal: mice fed the standard chow diet plus drinking water with intraperitoneal injection of D-galactose (120 mg/kg of BW); D-gal + VC: vitamin C (100 mg/kg of BW) plus intraperitoneal injection of D-galactose (120 mg/kg of BW); D-gal + NO: non-fermented soy milk (0.1 mL/10 g of BW) plus intraperitoneal injection of D-galactose (120 mg/kg of BW); D-gal + CQPC04: *L. fermentum* CQPC04-fermented soy milk (0.1 mL/10 g of BW) plus intraperitoneal injection of D-galactose (120 mg/kg of BW).

The aging of the skin is the most visible and obvious manifestation of organismal aging. H&E, Masson, and toluidine blue stains are usually used to observe pathological features of the skin. In the D-gal group, the skin dermis thickness (Figure 4A) and collagen fiber content (Figure 4B) were significantly lower, while the mast cells (Figure 4C) were higher as compared to the normal group. However, the above indicators were improved after the mice were treated with VC, non-fermented soy milk, and *L. fermentum* CQPC04-fermented soy milk, in which there was a more pronounced effect from the *L. fermentum* CQPC04-fermented soy milk as compared to the other groups.

### Results of Oxidative Indicators in Serum, Liver Tissue, and Brain Tissue

Anti-oxidant enzymes, MDA, GSH, and NO can reflect the oxidative stress levels of aging mice induced by D-galactose. Tables 2, 3 show that T-SOD, GSH-Px, GSH, and CAT levels significantly decreased, while MDA and NO obviously decreased after treatment with D-galactose (*p* < 0.05), which indicated a decrease in the anti-oxidative capacity of the mice. It is worth noting that *L. fermentum* CQPC04-fermented soy milk notably enhanced the levels of GSH-Px, T-SOD, GSH, and CAT, and reduced MDA and NO (*p* < 0.05). Additionally, there was no obvious difference in these indicators between the normal group and the D-gal + CQPC04 group (*p* > 0.05).

**Table 2:** Levels of oxidative aging indicators in serum of D-gal induced aging mice.

**Group**	**T-SOD**	**MDA**	**GSH-PX**	**GSH**	**CAT**	**NO**
	**(U/mL)**	**(nmol/mL)**	**(activity unit)**	**(mgGSH/L)**	**(U/mL)**	**(μmol/L)**
Normal	19.87 ± 6.44^a^	5.57 ± 2.14^b^	690.63 ± 185.83^a^	165.62 ± 23.43^a^	46.83 ± 10.44^a^	1.12 ± 0.56^d^
D-gal	11.56 ± 1.58^c^	47.23 ± 16.82^a^	415.00 ± 79.80^c^	109.97 ± 14.49^b^	26.58 ± 2.39^c^	8.65 ± 1.03^a^
D-gal + VC	12.50 ± 3.27^c^	16.51 ± 4.21^b^	448.81 ± 111.95^c^	125.29 ± 10.74^b^	27.99 ± 7.52^c^	6.18 ± 1.19^b^
D-gal + NO	13.80 ± 5.00^bc^	14.11 ± 5.56^b^	511.90 ± 77.41^bc^	152.00 ± 14.96^a^	36.46 ± 8.94^b^	6.72 ± 2.02^ab^
D-gal + CQPC04	17.60 ± 4.24^ab^	11.23 ± 3.31^b^	621.88 ± 86.25^ab^	167.73 ± 15.60^a^	41.01 ± 5.51^b^	4.16 ± 0.97^c^

*Values are presented as the mean ± standard deviation (n = 10/group). ^a−d^Mean values with different letters in the same column differ significantly (p <0.05) according to Duncan's significantly different test. D-gal: mice accepted an intraperitoneal injection of D-galactose [120 mg/(kg·bw)]; D-gal + VC: mice accepted oral administration of vitamin C [100 mg/(kg·bw)] and intraperitoneal injection of D-galactose [120 mg/(kg·bw)]; D-gal + NO: mice accepted non-fermented soy milk [0.1 mL/10(g·bw)] and intraperitoneal injection of D-galactose [120 mg/(kg·bw)]; D-gal + CQPC04: mice accepted L. fermentum CQPC04-fermented soy milk [0.1 mL/10(g·bw)] and intraperitoneal injection of D-galactose [120 mg/(kg·bw)]*.

**Table 3:** Levels of oxidative indicators in liver and brain of D-gal induced aging mice.

**Group**	**T-SOD**	**MDA**	**GSH-PX**	**GSH**	**CAT**	**NO**
	**(U/mgprot)**	**(nmol/mgprot)**	**(activity unit)**	**(mgGSH/gprot)**	**(U/mgprot)**	**(μmol/gprot)**
**Liver levels**
Normal	44.19 ± 7.93^a^	3.00 ± 0.99^b^	866.50 ± 53.79^a^	9.22 ± 1.07^a^	105.84 ± 4.31^ab^	0.023 ± 0.009^c^
D-gal	37.54 ± 1.37^d^	9.19 ± 0.95^a^	658.35 ± 144.34^c^	7.21 ± 0.41^c^	99.27 ± 3.95^c^	0.038 ± 0.015^a^
D-gal + VC	39.83 ± 4.54^c^	2.52 ± 1.14^b^	790.46 ± 47.55^b^	7.77 ± 0.55^bc^	102.98 ± 2.73^bc^	0.030 ± 0.008^ab^
D-gal + NO	38.76 ± 3.17^c^	3.54 ± 1.8^b^	804.46 ± 87.35^b^	8.02 ± 0.60^b^	102.57 ± 2.40^bc^	0.032 ± 0.010^ab^
D-gal + CQPC04	42.99 ± 3.84^b^	2.56 ± 0.89^b^	819.40 ± 100.54^a^	9.21 ± 1.14^a^	106.87 ± 3.14^a^	0.012 ± 0.007^bc^
**Brain levels**
Normal	8.98 ± 0.61^a^	1.52 ± 0.16^c^	57.68 ± 11.49^a^	10.44 ± 0.58^a^	28.89 ± 8.15^a^	0.005 ± 0.004^c^
D-gal	7.54 ± 0.68^c^	2.05 ± 0.38^a^	29.33 ± 12.60^c^	8.85 ± 0.42^c^	18.72 ± 2.04^c^	0.040 ± 0.008^a^
D-gal + VC	7.52 ± 0.63^c^	1.92 ± 0.21^ab^	39.20 ± 8.89^bc^	9.35 ± 0.58^bc^	22.00 ± 4.47^bc^	0.034 ± 0.012^ab^
D-gal + NO	7.98 ± 0.60^bc^	1.81 ± 0.22^abc^	41.96 ± 5.48^b^	9.42 ± 0.50^bc^	23.48 ± 4.24^abc^	0.030 ± 0.009^ab^
D-gal + CQPC04	8.29 ± 0.51^b^	1.65 ± 0.36^bc^	44.80 ± 7.05^b^	10.59 ± 0.68^a^	29.92 ± 6.96^a^	0.026 ± 0.004^b^

*Values are presented as the mean ± standard deviation (n = 10/group). ^a−d^Mean values with different letters in the same column differ significantly (p <0.05) according to Duncan's significantly different test. D-gal: mice accepted an intraperitoneal injection of D-galactose [120 mg/(kg·bw)]; D-gal + VC: mice accepted oral administration of vitamin C [100 mg/(kg·bw)] and intraperitoneal injection of D-galactose [120 mg/(kg·bw)]; D-gal + NO: mice accepted non-fermented soy milk [0.1 mL/10(g·bw)] and intraperitoneal injection of D-galactose [120 mg/(kg·bw)]; D-gal + CQPC04: mice accepted L. fermentum CQPC04-fermented soy milk [0.1 mL/10(g·bw)] and intraperitoneal injection of D-galactose [120 mg/(kg·bw)]*.

### Results of Oxidative Indicators in Skin Tissue

**Figure 5:**
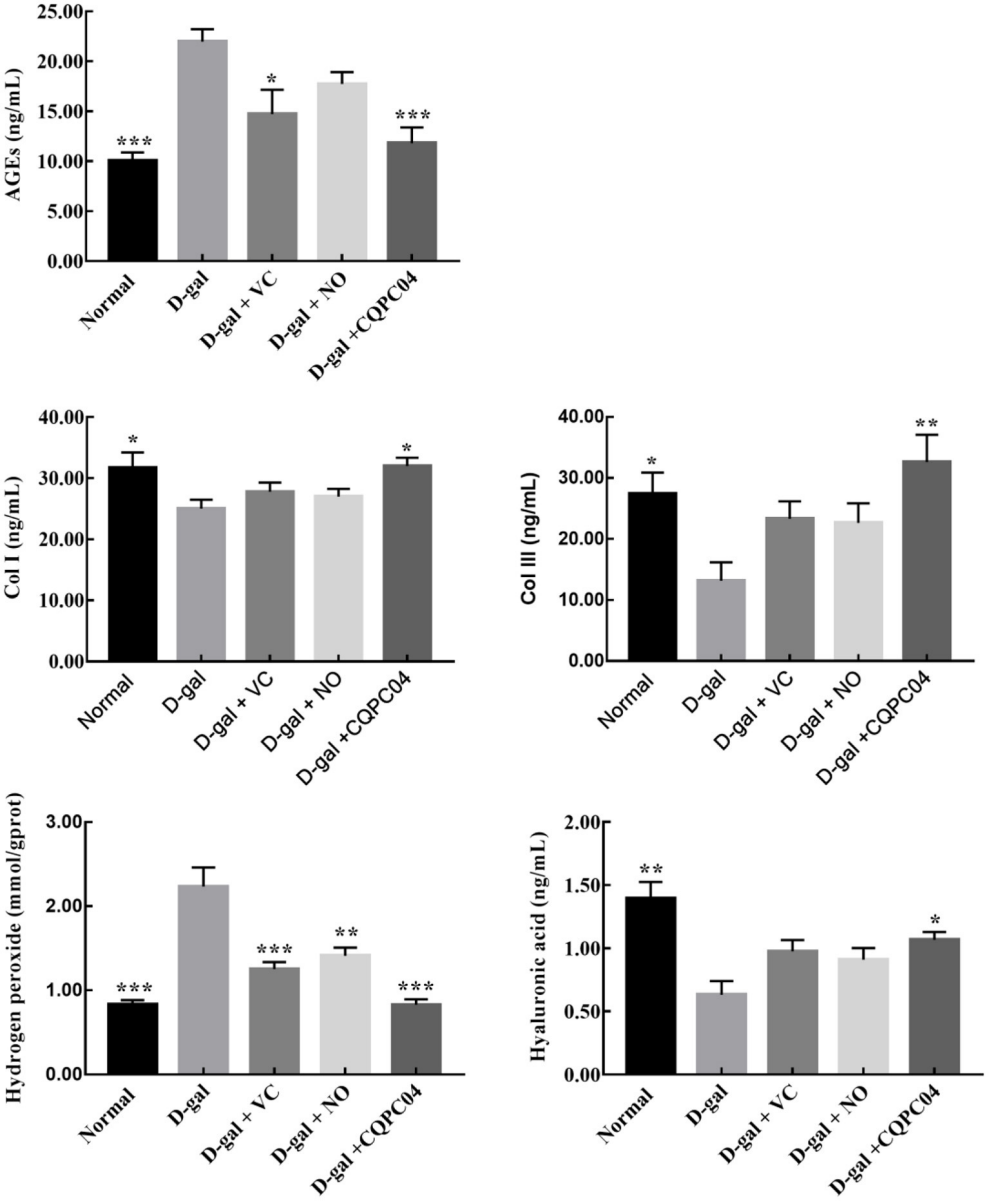
Levels of Col I, Col III, AGEs, hyaluronic acid and hydrogen peroxide in skin tissue. D-gal: mice fed the standard chow diet plus drinking water with intraperitoneal injection of D-galactose (120 mg/kg of BW); D-gal + VC: vitamin C (100 mg/kg of BW) plus intraperitoneal injection of D-galactose (120 mg/kg of BW); D-gal + NO: non-fermented soy milk (0.1 mL/10 g of BW) plus intraperitoneal injection of D-galactose (120 mg/kg of BW); D-gal + CQPC04: *L. fermentum* CQPC04-fermented soy milk (0.1 mL/10 g of BW) plus intraperitoneal injection of D-galactose (120 mg/kg of BW). *p < 0.05, **p < 0.01, and ***p < 0.001 compared to D-gal group.

In order to further evaluate the effect of *L. fermentum* CQPC04-fermented soy milk on D-galactose-induced aging mice, we measured the amounts of AGEs, hyaluronic acid, Col I, hydrogen peroxide, and Col III in skin tissues (Figure 5). We found that the levels of Col I, hyaluronic acid, and Col III were the highest, and AGEs and hydrogen peroxide were the lowest in the normal group, but these indicators were absolutely in reverse in the D-gal group. In addition, VC, non-fermented soy milk, and *L. fermentum* CQPC04-fermented soy milk increased levels of Col I, hyaluronic acid, and Col III, but reduced the level of hydrogen peroxide and AGEs. These indicators in the D-gal + CQPC04 group were similar to those of the normal group, which demonstrated that *L. fermentum* CQPC04 has ability to delay the aging process in the skin.

### mRNA Expression Levels of Genes Related to Nrf2 in the Liver and Spleen

**Figure 6:**
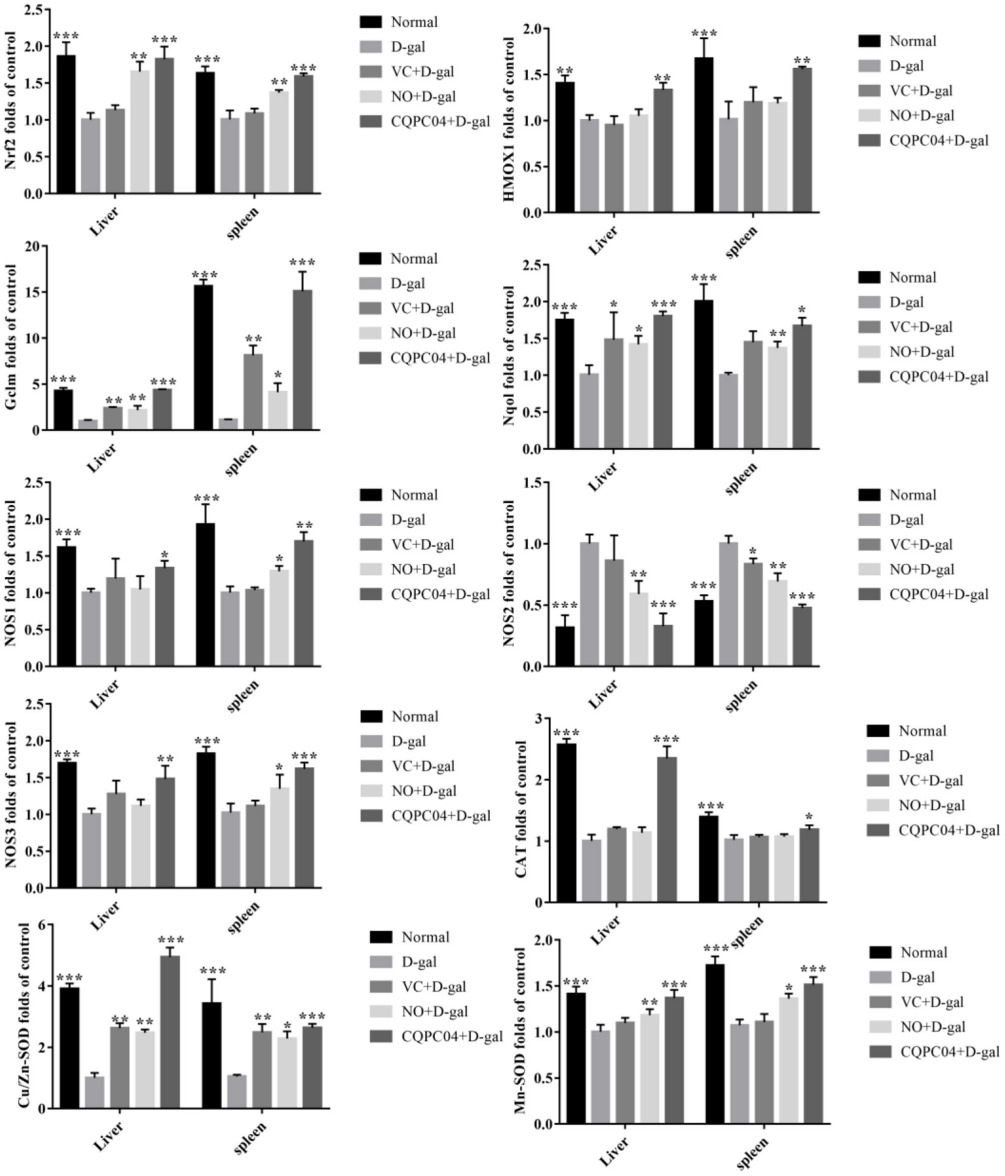
mRNA expression of Nrf2, HMOX1, Nqol, Gclm, NOS1, NOS2, NOS3, CAT, Cu/Zn-SOD, and Mn-SOD in the liver and spleen. D-gal: mice fed the standard chow diet plus drinking water with intraperitoneal injection of D-galactose (120 mg/kg of BW); D-gal + VC: vitamin C (100 mg/kg of BW) plus intraperitoneal injection of D-galactose (120 mg/kg of BW); D-gal + NO: non-fermented soy milk (0.1 mL/10 g of BW) plus intraperitoneal injection of D-galactose (120 mg/kg of BW); D-gal + CQPC04: *L. fermentum* CQPC04-fermented soy milk (0.1 mL/10 g of BW) plus intraperitoneal injection of D-galactose (120 mg/kg of BW). *p < 0.05, **p < 0.01, and ***p < 0.001 compared to D-gal group.

The RT-qPCR results indicate that the expression trend of related genes in the liver and spleen is consistent (Figure 6). The expression levels of *Nrf2, HMOX1, Nqol, NOS1, NOS3, CAT, Cu/Zn-SOD*, and *Mn-SOD* decreased, but *NOS2* and *Gclm* increased after mice were treated with D-galactose, in which the greatest amount of change in the above indicators occurred in the D-gal group. VC, non-fermented soy milk, and *L. fermentum* CQPC04-fermented soy milk upregulated the expression of *Nrf2, HMOX1, Nqol, NOS1, NOS3, CAT, Cu/Zn-SOD*, and *Mn-SOD*, and downregulated the expression of *NOS2* and *Gclm*. Compared with the D-gal + VC and D-gal + NO groups, the expression of the *Nrf2, HMOX1, Nqol, NOS1, NOS3, CAT, Cu/Zn-SOD*, and *Mn-SOD* genes in the liver and spleen tissues of mice in the D-gal + CQPC04 group was significantly increased, while the expression of the *NOS2* and Gclm genes was significantly decreased, and the results were similar to those of the normal group (*p* > 0.05).

### mRNA Expression Levels of Oxidative Aging Genes in the Skin

**Figure 7:**
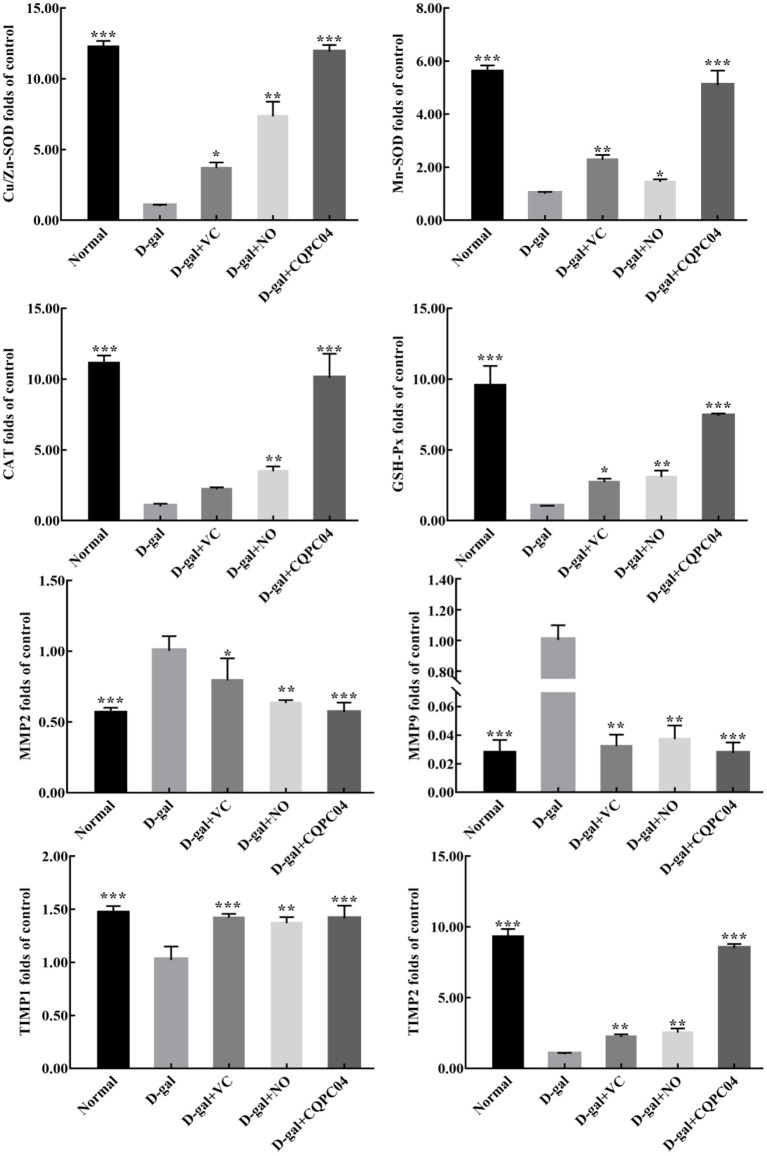
mRNA expression of Cu/Zn-SOD, Mn-SOD, CAT, GSH-Px, MMP2, MMP9, TIMP1, and TIMP2 in skin. D-gal: mice fed the standard chow diet plus drinking water with intraperitoneal injection of D-galactose (120 mg/kg of BW); D-gal + VC: vitamin C (100 mg/kg of BW) plus intraperitoneal injection of D-galactose (120 mg/kg of BW); D-gal + NO: non-fermented soy milk (0.1 mL/10 g of BW) plus intraperitoneal injection of D-galactose (120 mg/kg of BW); D-gal + CQPC04: *L. fermentum* CQPC04-fermented soy milk (0.1 mL/10 g of BW) plus intraperitoneal injection of D-galactose (120 mg/kg of BW). *p < 0.05, **p < 0.01, and ***p < 0.001 compared to D-gal group.

Antioxidant enzymes and matrix metalloproteinases are important indicators that can be used to evaluate the degree of skin aging. As shown in Figure 7, the mRNA expression of *Cu/Zn-SOD, Mn-SOD, CAT, GSH*-Px, tissue inhibitor of metalloproteinase (*TIMP*)1, and TIMP2 was the lowest, while that of matrix metalloproteinase (*MMP*)2 and MMP9 was the highest in the D-gal group. Compared with the D-gal group, the expression levels of *Cu/Zn-SOD, Mn-SOD, CAT, GSH-Px, TIMP1*, and *TIMP2* increased, but *MMP2* and *MMP9* decreased in the D-gal + VC, D-gal + NO, and D-gal + CQPC04 groups. It is worth noting that the mouse skin in the D-gal + CQPC04 group possessed the highest mRNA expression levels of *Cu/Zn-SOD, Mn-SOD, CAT, GSH*-*Px, TIMP1*, and *TIMP2*, and the lowest expression levels of *MMP2* and *MMP9* except for the normal group, which demonstrated that *L. fermentum* CQPC04-fermented soy milk reduced the oxidant aging of skin and decreased the degradation of collagen fibers.

## Discussion

Soy milk fermented with lactic acid bacteria is beneficial to human health because of the bioactive components in the milk that play important roles. Studies have proved that using lactic acid bacteria to ferment soy milk can convert the conjugated isoflavones and proteins into aglycone isoflavones and bioactive peptides, respectively, which increases the bioavailability of these substances in the human intestinal tract (19). At present, many studies have verified that fermented soy milk possesses anti-obesity, anti-cancer, anti-hyperglycemic, and anti-hypertensive action, and can also prevent cardiovascular disease, but few studies have reported that fermented soy milk confers an anti-aging effect (20). Therefore, we chose the strain named *L. fermentum* CQPC04 to ferment soy milk and evaluated the changes in isoflavones and peptides, as well as explored the antioxidant effect of this fermented soy milk due to its antioxidant action.

Soybean products, such as soy milk, contain a large amount of protein and conjugated isoflavones, and fermentation by microoganisms can transform the above components into small bioactive peptides and aglycone isoflavones that play a decisive role in the total antioxidant capacity of fermented soy milk (21). Previous studies claimed that the content and composition of isoflavones and peptides were different in fermented soybean products that were fermented with different types of microoganisms (22). One previous research found that the free glycosides in fermented soymilk with *L. plantarum* CQPC02 (7.59 μg/mL) were obviously higher than those of non-fermented soymilk (4.01 μg/mL) and fermented soymilk with *Lactobacillus bulgaricus* (4.05 μg/mL) (23). Another study indicated that the isoflavones transformation capacity of different lactic acid bacteria was significantly different, in which the aglycones in *E. faecium* 3 fermented soy milk could be increased to 71% of total isoflavone (24). Our experimental results revealed that there was a significant increase in the amounts of peptides and aglycone isoflavones in soy milk after being fermented with *L. fermentum* CQPC04, which indicated that *L. fermentum* CQPC04 can promote the generation of bioactive components and increase the anti-oxidant capacity of soy milk.

With increasing age or as the aging process accelerates, the body's organs and tissues will gradually undergo changes such as atrophy and functional degradation. Our study found that the organ indexes of the liver and spleen was significantly decreased, and the histopathological morphology of the liver, spleen, and skin was damaged to varying degrees in mice, which showed that the anti-oxidative aging model was successfully established (25). In contrast, the above indicators were significantly improved after the mice were treated with *L. fermentum* CQPC04-fermented soy milk, and resulted in the high organ index of the liver and spleen, a return to the normal structure of the liver, spleen, and skin, as well as increased collagen fibers and few mast cells in the skin.

A previous study reported that natto suppressed fatty acid synthesis, promoted fatty acid catabolism, and maintained the normal physiology of the liver (26). Another study revealed that *L. sakei* K040706, which is from traditional Korean fermented soybean paste, increased the spleen indices of cyclophosphamide-induced immunosuppressed mice (27). In addition, the ability of isoflavones and soy peptides to increase collagen in the skin and reduce photo-aging of the skin had also been proved (28, 29). Based on these findings, our preliminary conclusion is that the antioxidant effect of *L. fermentum* CQPC04-fermented soy milk on organs or tissues may be related to the synergistic antioxidant capacity of *L. fermentum* CQPC04, isoflavones, and peptides.

Antioxidants, such as Cu/Zn-SOD, Mn-SOD, T-SOD, GSH-Px, CAT, and GSH, play a critical role in defending against free radical damage to the human body (30). In addition, MDA and NO are also critical indicators for evaluating oxidative stress (31). It is well-known that fermentation can greatly affect the amounts of isoflavone aglycones and peptides, and the antioxidant activity in soy milk (32). In the present study, *L. fermentum* CQPC04-fermented soy milk increased the levels of T-SOD, GSH-Px, CAT, and GSH, but reduced MDA and NO levels in serum, and liver and brain tissues. It also upregulated the mRNA expression of *Cu/Zn-SOD, Mn-SOD, GSH-Px*, and *CAT* in liver, spleen, and skin tissues. A stereological study revealed that *L. casei-*fermented soy milk significantly increased blood levels of CAT and the total antioxidant capacity (TAC) (33). A previous study demonstrated that the *in vitro* and *in vivo* antioxidant activity, which were related to the isoflavone and peptide content, significantly increased after soy meal was fermented by *Bacillus amyloliquefaciens* SWJS22. The specific manifestation was that the DPPH radical scavenging capacity and reducing power were enhanced, and the T-SOD, CAT, GSH-Px, and TAC activities increased, while there was decreased MDA in the liver and serum of D-galactose-induced oxidative aging mice (34).

Long-term oxidative stress reactions will generate a large amount of ROS, which can cause oxidative damage to the skin (35). Collagen, AGEs, HA, hydrogen peroxide, MMPs, and TIMPs play important roles in the aging process of the skin (36). Collagen I and collagen III are the main structural proteins of the extracellular matrix (ECM), and are important in maintaining young-looking skin. They will be hydrolyzed by MMP2 and MMP9 when the skin is exposed to UVB or other oxidative stress (37). MMP inhibitors, such as TIMP1 and TIMP2, are mainly responsible for degrading metalloproteinases and indirectly delay the oxidative aging of skin (38). In addition, a previous study claimed that accumulation of AGEs modified the activities of MMPs and resulted in the occurrence of EMC degradation (39). High levels of H_2_O_2_ will cause cytotoxicity to the skin, and lower amounts of HA can lead to reduced moisture, the presence of roughness and wrinkles, and a loss of skin elasticity (40, 41).

In the current study, we found that *L. fermentum* CQPC04-fermented soy milk increased Col I, Col III, and HA, but decreased H_2_O_2_ and AGEs in skin tissue. It also upregulated the mRNA expression of TIMP1 and TIMP2, and decreased the expression of MMP2 and MMP9 in skin tissue. These results indicate that *L. fermentum* CQPC04-fermented soy milk is beneficial to maintain the youthful and healthy state of the skin, and it can provide a new dietary choice for people who are interested in skin care (42).

Except for the related gene expression changes in skin tissue, we also found that other genes were modified by *L. fermentum* CQPC04-fermented soy milk in liver and spleen tissues, that is, the mRNA expression of Nrf2, HMOX1, Nqo1, NOS1, and NOS3 increased, while NOS2 and Gclm expression decreased. The Nrf2/HMOX1 signaling pathway is responsible for the expression of hundreds of antioxidant genes or enzymes. A previous study reported that the mRNA expression of Nrf2 and HMOX1 decreased, while the expression of Gclm increased in D-galactose-induced aging chickens, which is consistent with our results (43). The activation of Nrf2 is usually accompanied by high expression of the Nqo1 gene, which plays an important role in the cellular redox state (44). In addition, NOS1, NOS2, and NOS3 are all oxidative genes related to Nrf2/HMOX1 signaling (45). Soybean peptides are able to upregulate the enzymatic activities of SOD, CAT, and GSH-Px, and reduce MDA by activating the gene expression of Nrf2, and we also found similar results in the present study (46). Another study revealed that soybean isoflavones reduced the oxidative stress and inflammatory state of patients with ischemic stroke by activating Nrf2 (47). Based on these studies, we concluded that *L. fermentum* CQPC04-fermented soy milk conferred an antioxidant effect that may be related to the activation of Nrf2, and it can be used as a functional food.

## Conclusion

In the present study, we found that *L. fermentum* CQPC04 promoted the conversion of soybean proteins and conjugate isoflavones into bioactive peptides and aglycone isoflavones, respectively. In addition, *L. fermentum* CQPC04-fermented soy milk clearly increased the antioxidant capacity of mice treated with D-galactose, and the mechanism may be related to the activation of peptides and aglycone isoflavones by Nrf2/HMOX1. The experimental results can provide a reference and data support for the food industry to develop new functional fermented soy milk, and can also provide new ideas for research regarding the delay of oxidative response.

## Data Availability Statement

The original contributions presented in the study are included in the article/Supplementary Material, further inquiries can be directed to the corresponding author/s.

## Ethics Statement

The animal study was reviewed and approved by the Chongqing Functional Food Collaborative Innovation Center (IACUC Number: 201905001B) and also complied with the 2010/63/EU directive.

## Author Contributions

XZho and H-hD performed the majority of the experiments and wrote the manuscript. MJ, CZ, and YD contributed to the data analysis. XZha and XL designed and supervised the study and checked the final manuscript. All authors contributed to the article and approved the submitted version.

## Conflict of Interest

The authors declare that the research was conducted in the absence of any commercial or financial relationships that could be construed as a potential conflict of interest.

## Publisher's Note

All claims expressed in this article are solely those of the authors and do not necessarily represent those of their affiliated organizations, or those of the publisher, the editors and the reviewers. Any product that may be evaluated in this article, or claim that may be made by its manufacturer, is not guaranteed or endorsed by the publisher.
