# Supplementary material for: Antioxidant Effect of Lactobacillus fermentum CQPC04-Fermented Soy Milk on D-Galactose-Induced Oxidative Aging Mice
Source: Front Nutr. 2021 Aug 27;8:727467. doi: 10.3389/fnut.2021.727467 (PMC8429822; doi:10.3389/fnut.2021.727467)
Supplement: Supplementary file 1 [file Table_1.DOCX]

Complementary materials


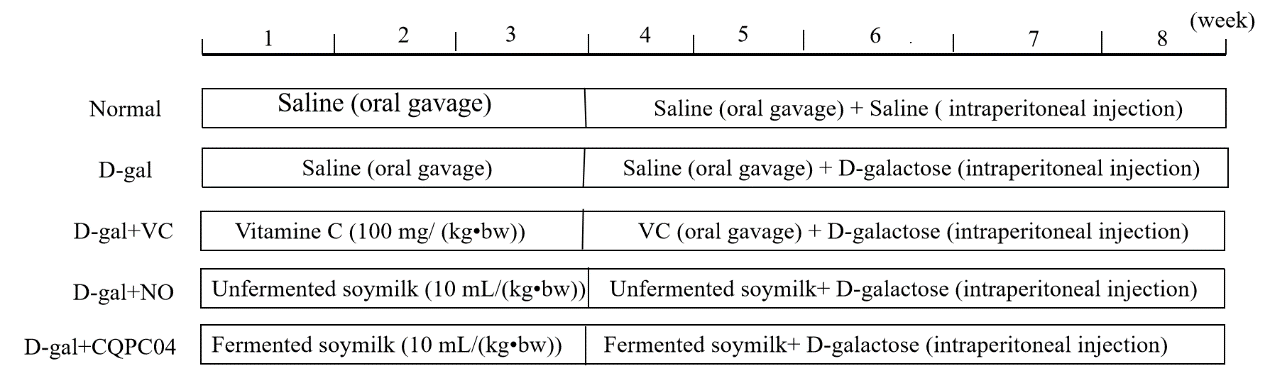


**Figure S1.** Grouping and treatment of mice. D-gal: mice fed the standard chow diet plus drinking water with intraperitoneal injection of D-galactose (120 mg/kg of BW); D-gal + VC: vitamin C (100 mg/kg of BW) plus intraperitoneal injection of D-galactose (120 mg/kg of BW); D-gal + NO: non-fermented soy milk (0.1 mL/10g of BW) plus intraperitoneal injection of D-galactose (120 mg/kg of BW); D-gal + CQPC04: L. fermentum CQPC04-fermented soy milk (0.1 mL/10 g of BW) plus intraperitoneal injection of D-galactose (120 mg/kg of BW).
