# Supplementary figures and images for: Antioxidant Effect of Lactobacillus fermentum CQPC04-Fermented Soy Milk on D-Galactose-Induced Oxidative Aging Mice
Source: Front Nutr. 2021 Aug 27;8:727467. doi: 10.3389/fnut.2021.727467 (PMC8429822; doi:10.3389/fnut.2021.727467)

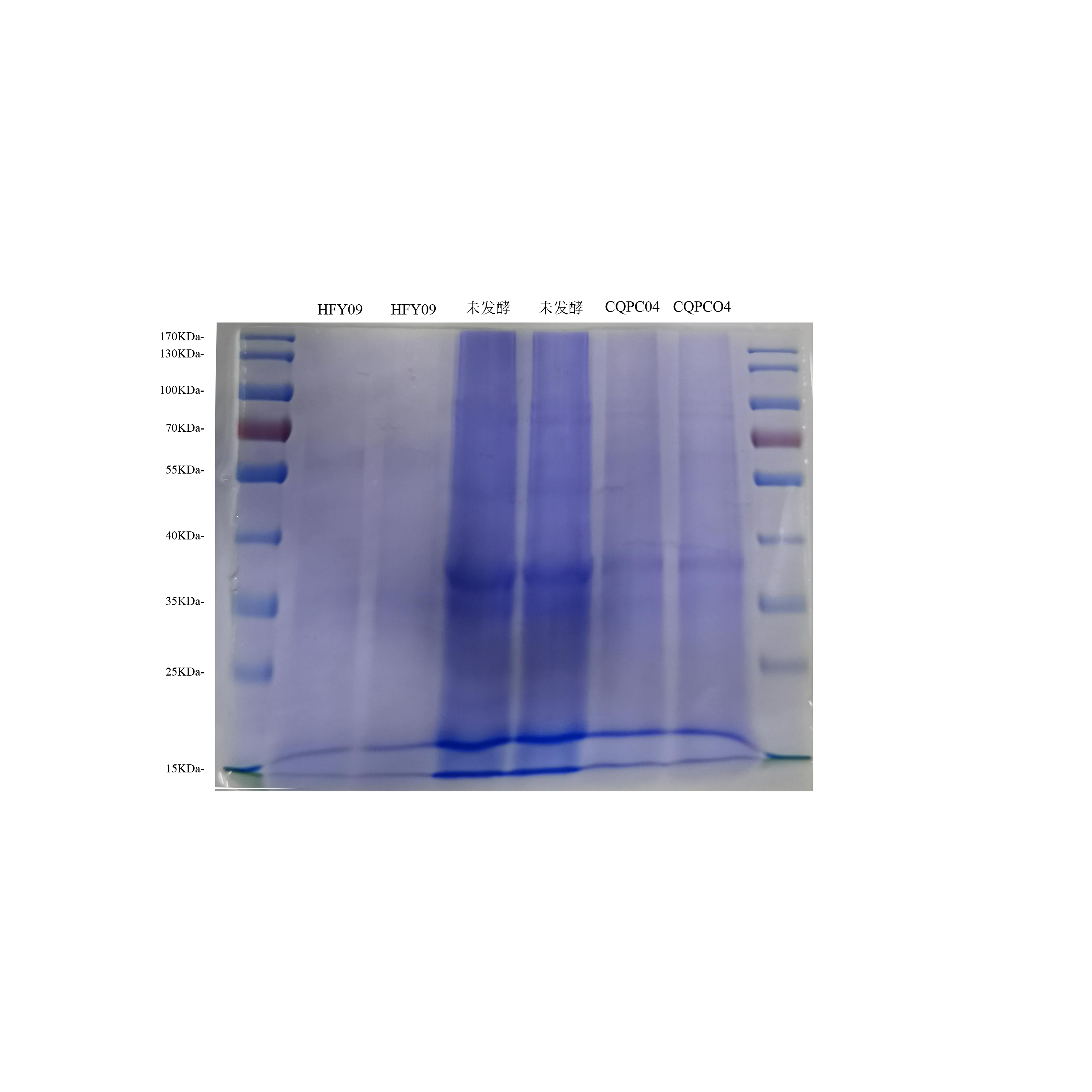

Supplement: Supplementary file 2 [file Data_Sheet_1.ZIP › Protein mass spectrometry.tif]

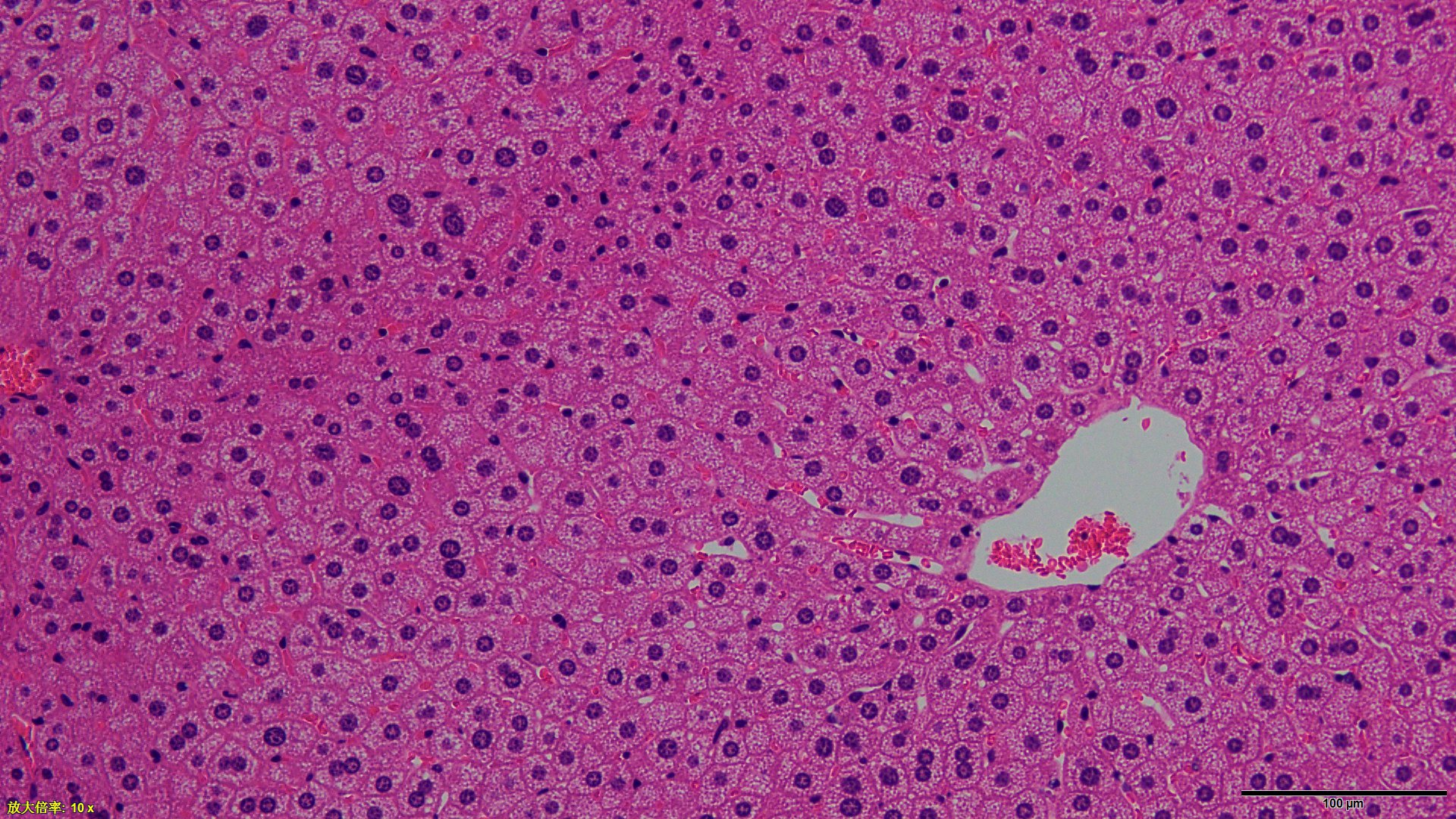

Supplement: Supplementary file 2 [file Data_Sheet_1.ZIP › SECTION/LIVER/CQPC04.jpg]

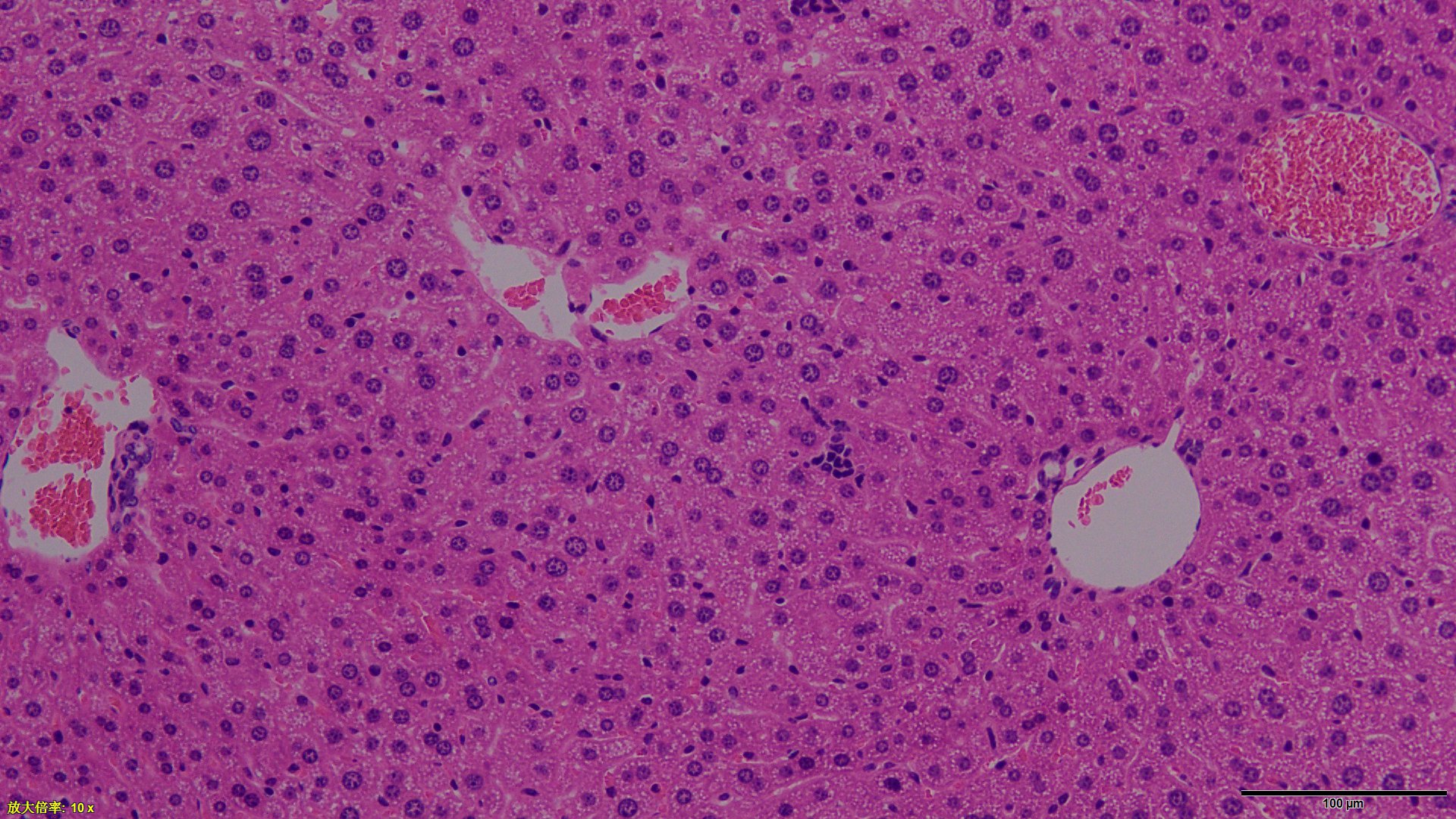

Supplement: Supplementary file 2 [file Data_Sheet_1.ZIP › SECTION/LIVER/NO.jpg]

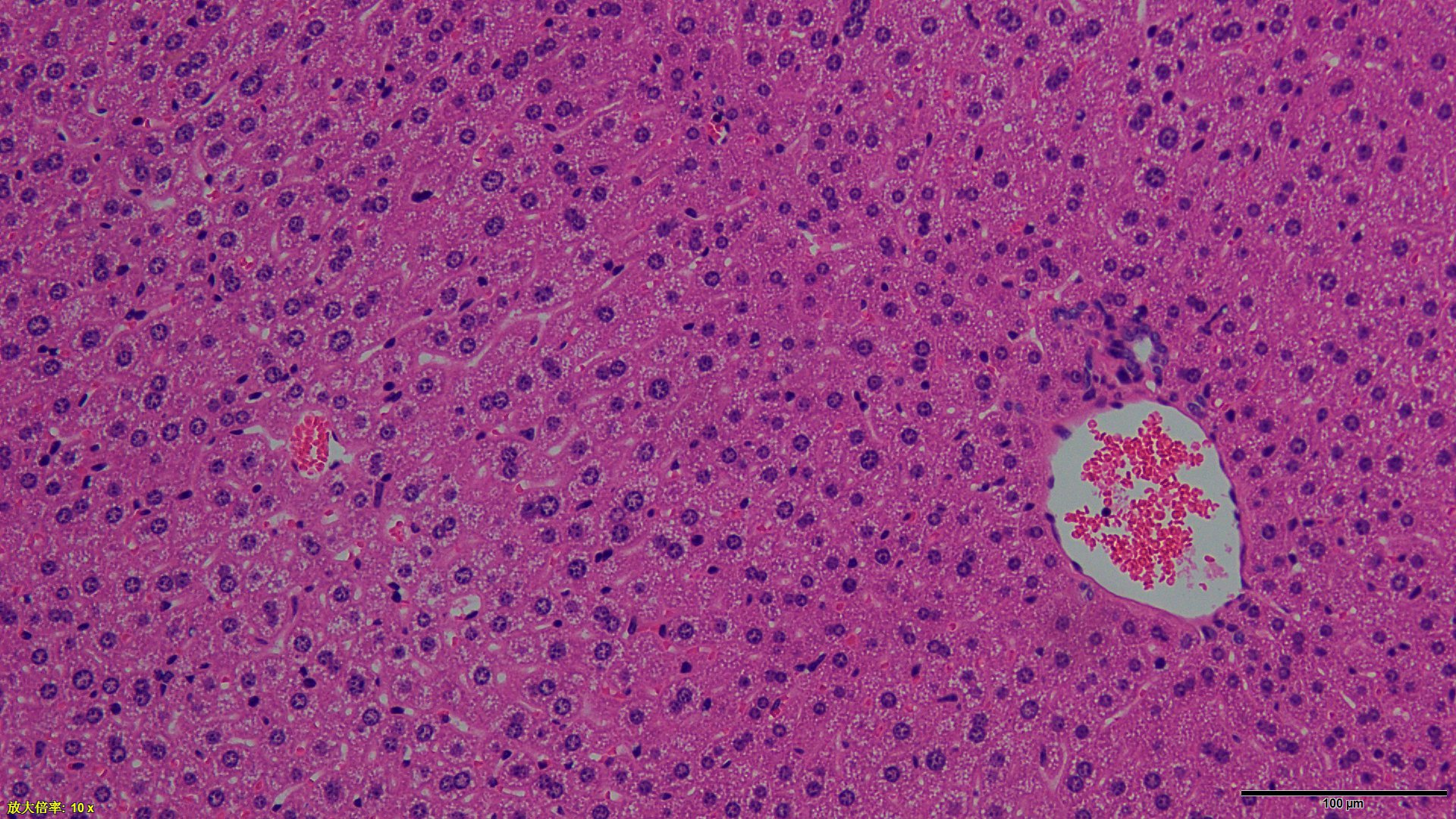

Supplement: Supplementary file 2 [file Data_Sheet_1.ZIP › SECTION/LIVER/VC.jpg]

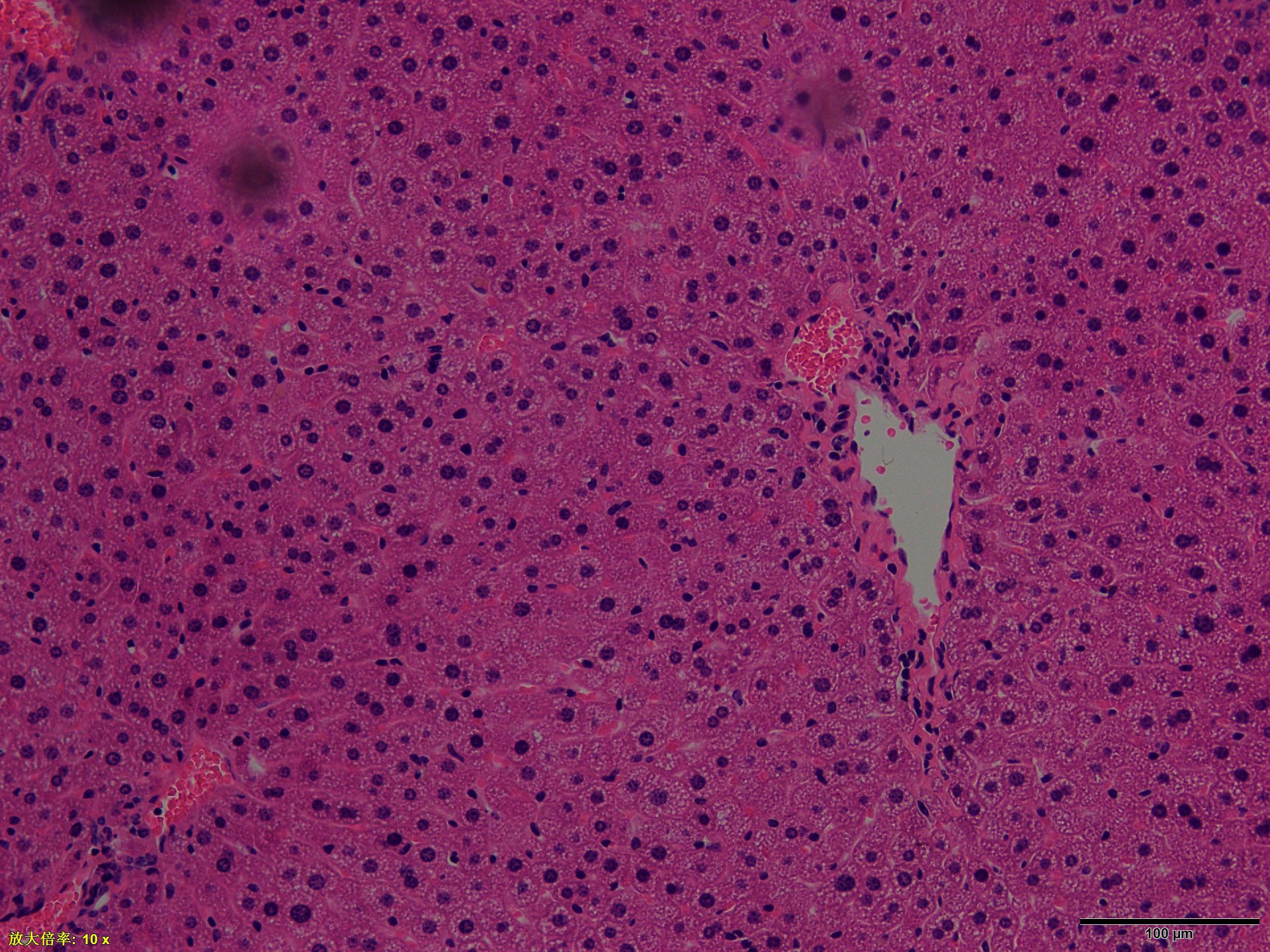

Supplement: Supplementary file 2 [file Data_Sheet_1.ZIP › SECTION/LIVER/模型.jpg]

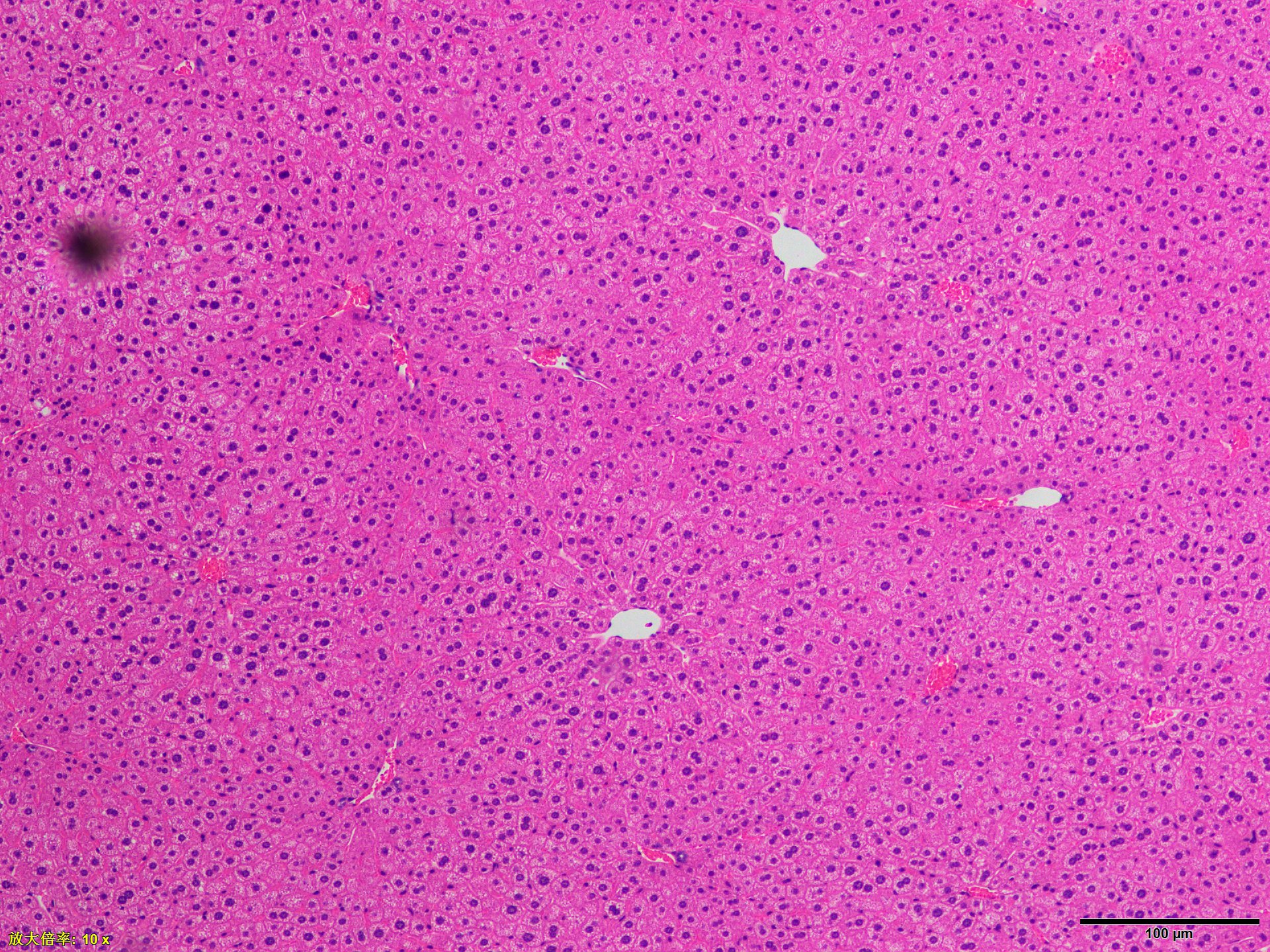

Supplement: Supplementary file 2 [file Data_Sheet_1.ZIP › SECTION/LIVER/正常.jpg]

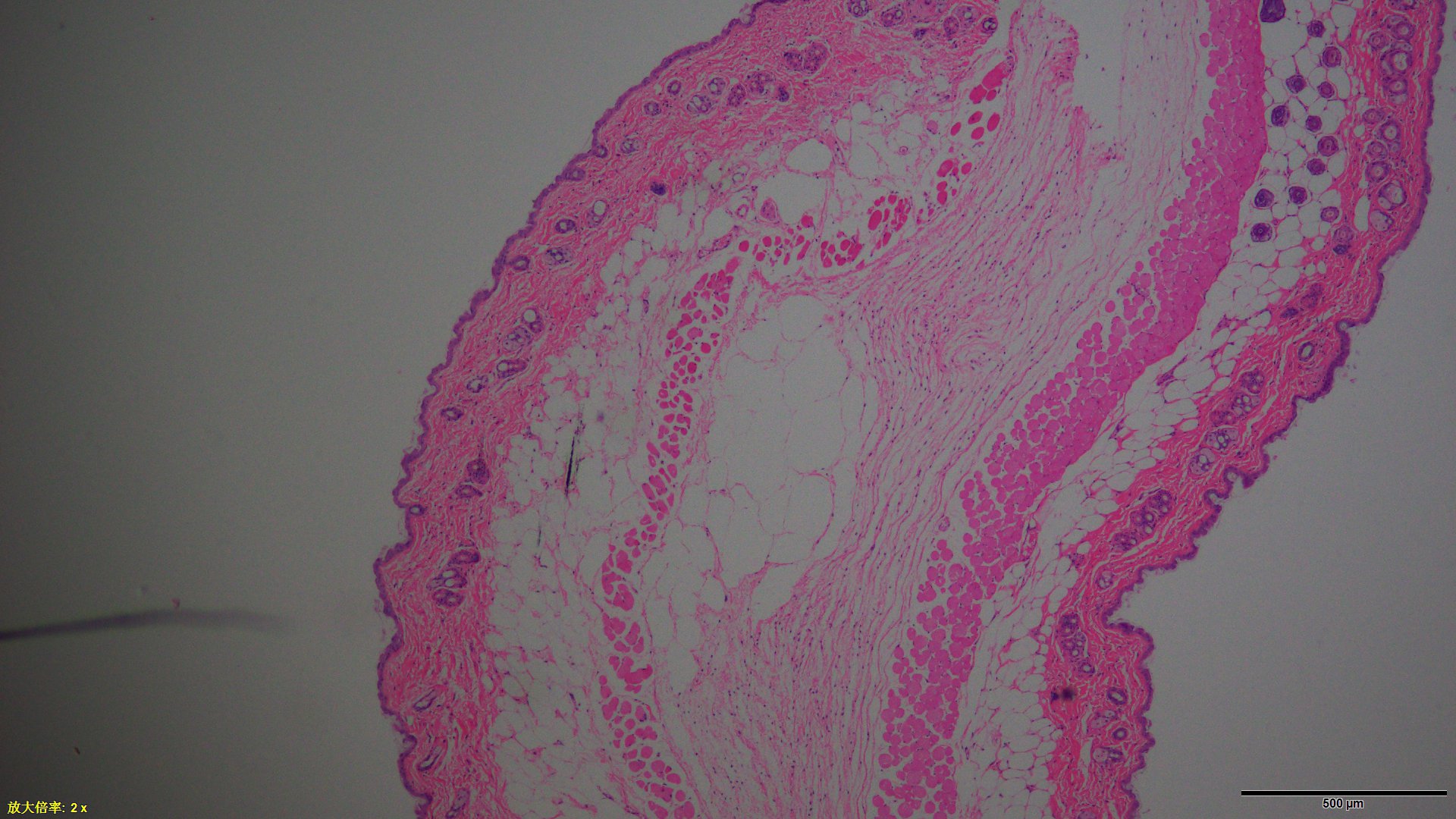

Supplement: Supplementary file 2 [file Data_Sheet_1.ZIP › SECTION/SKIN/HE/CQPC04.jpg]

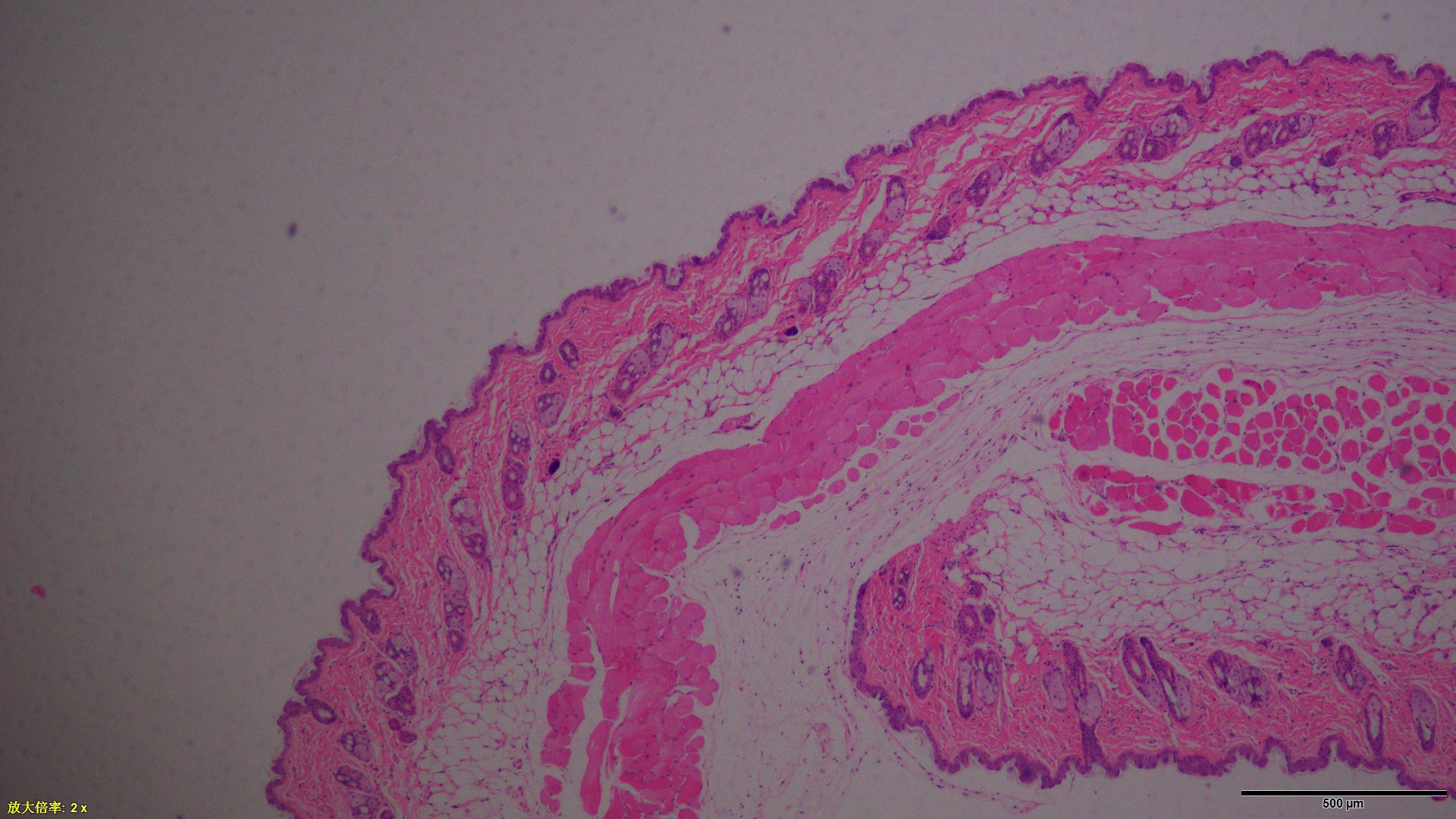

Supplement: Supplementary file 2 [file Data_Sheet_1.ZIP › SECTION/SKIN/HE/NO.jpg]

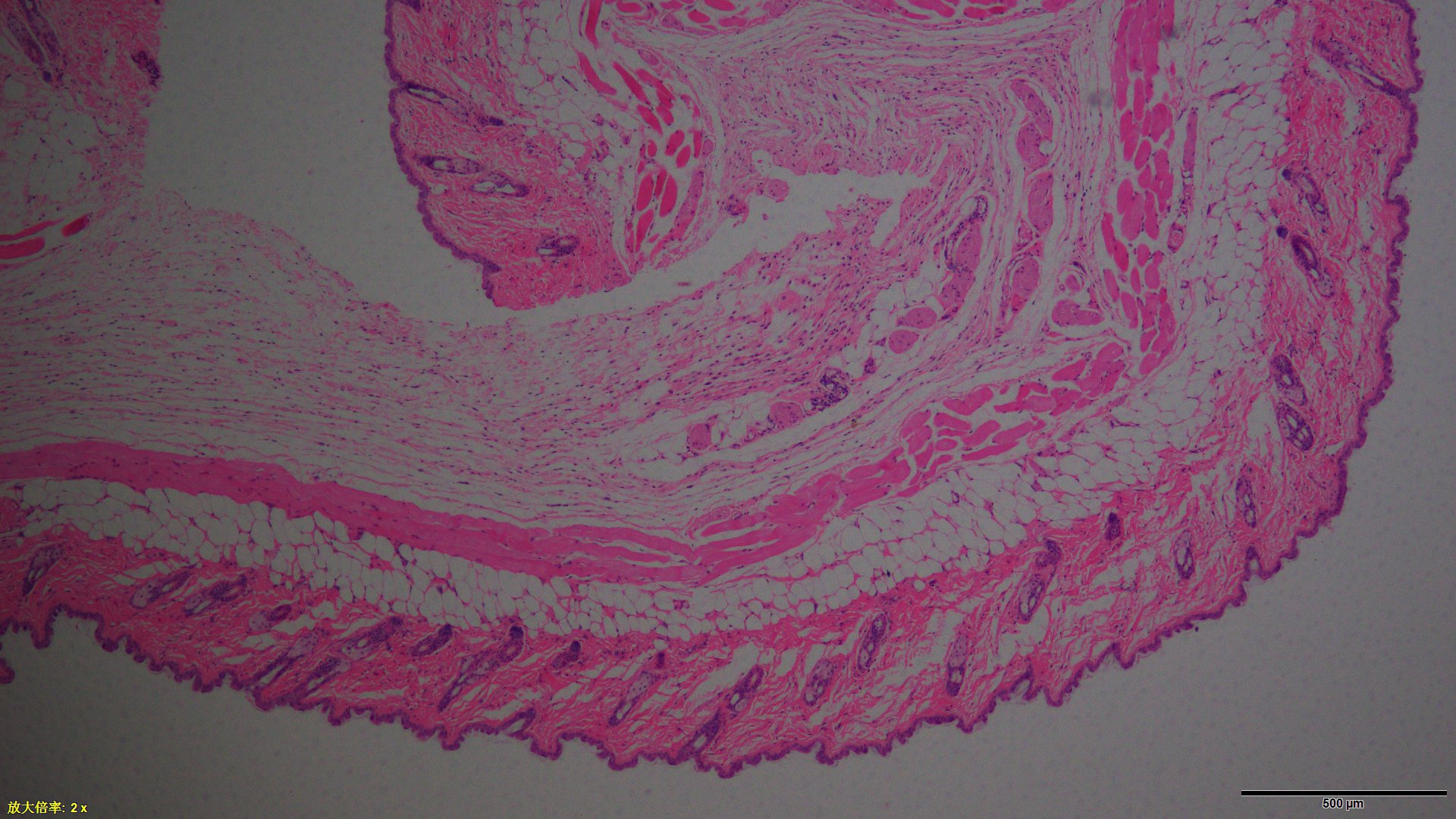

Supplement: Supplementary file 2 [file Data_Sheet_1.ZIP › SECTION/SKIN/HE/VC.jpg]

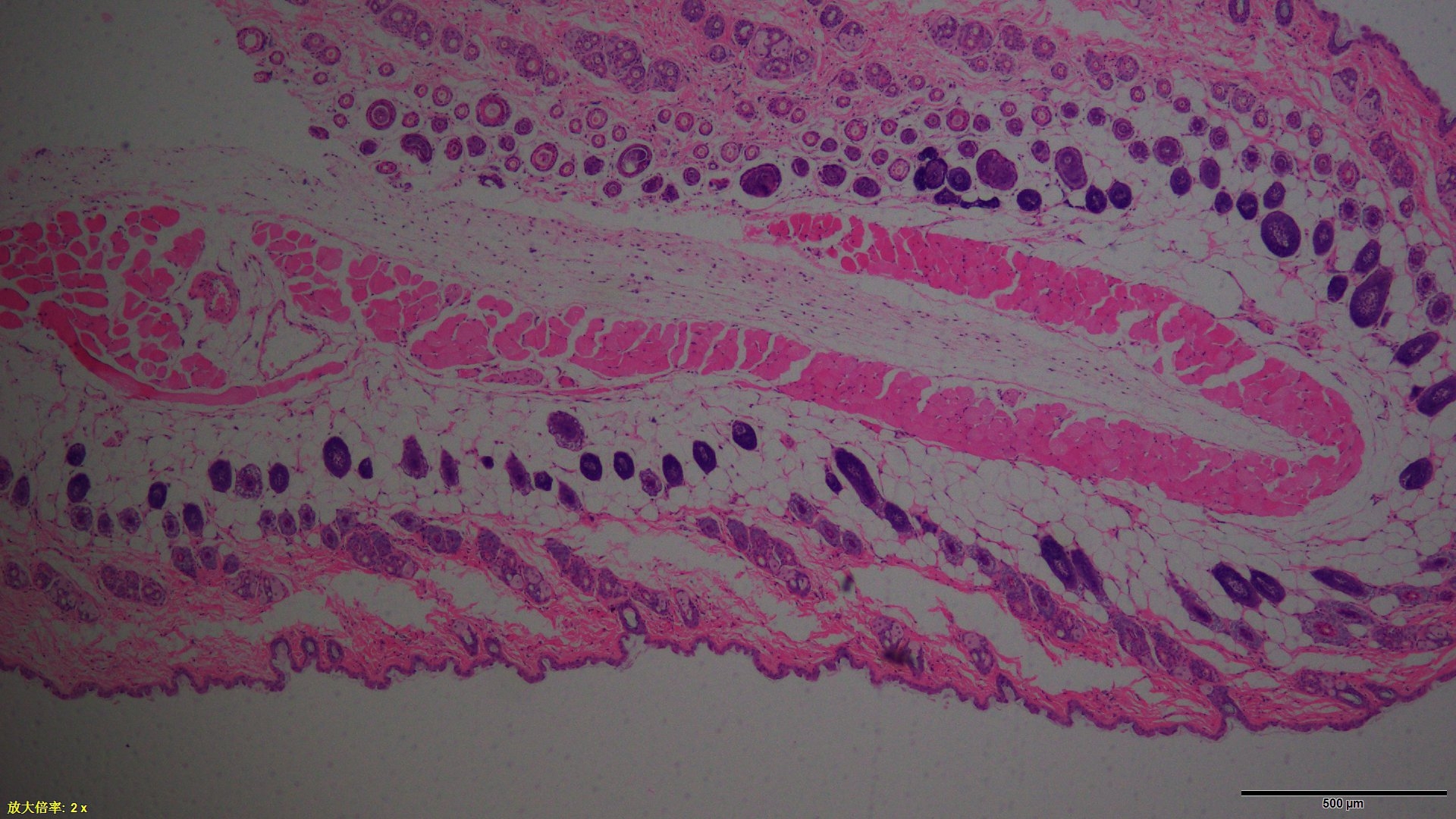

Supplement: Supplementary file 2 [file Data_Sheet_1.ZIP › SECTION/SKIN/HE/模型.jpg]

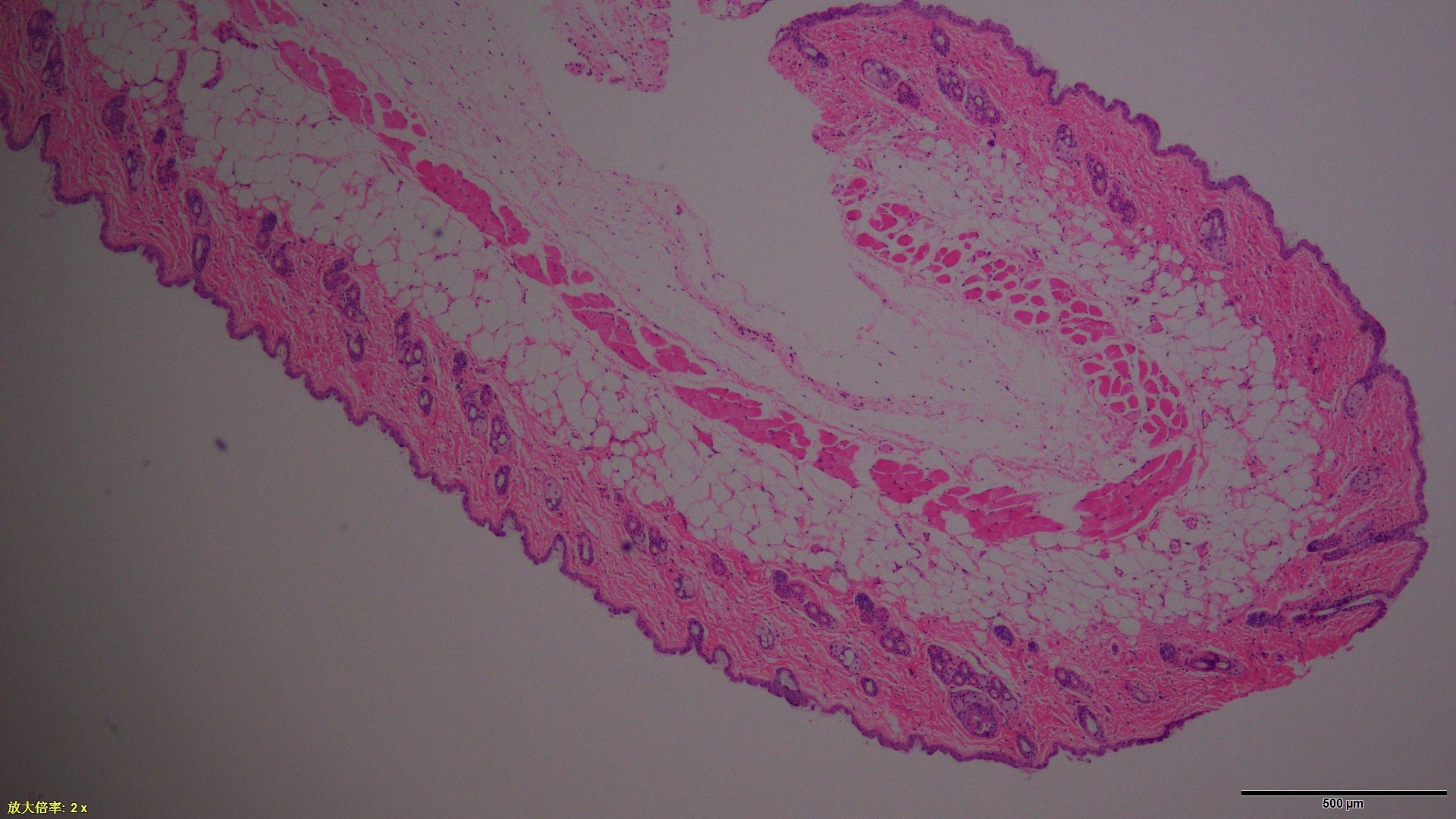

Supplement: Supplementary file 2 [file Data_Sheet_1.ZIP › SECTION/SKIN/HE/正常.jpg]

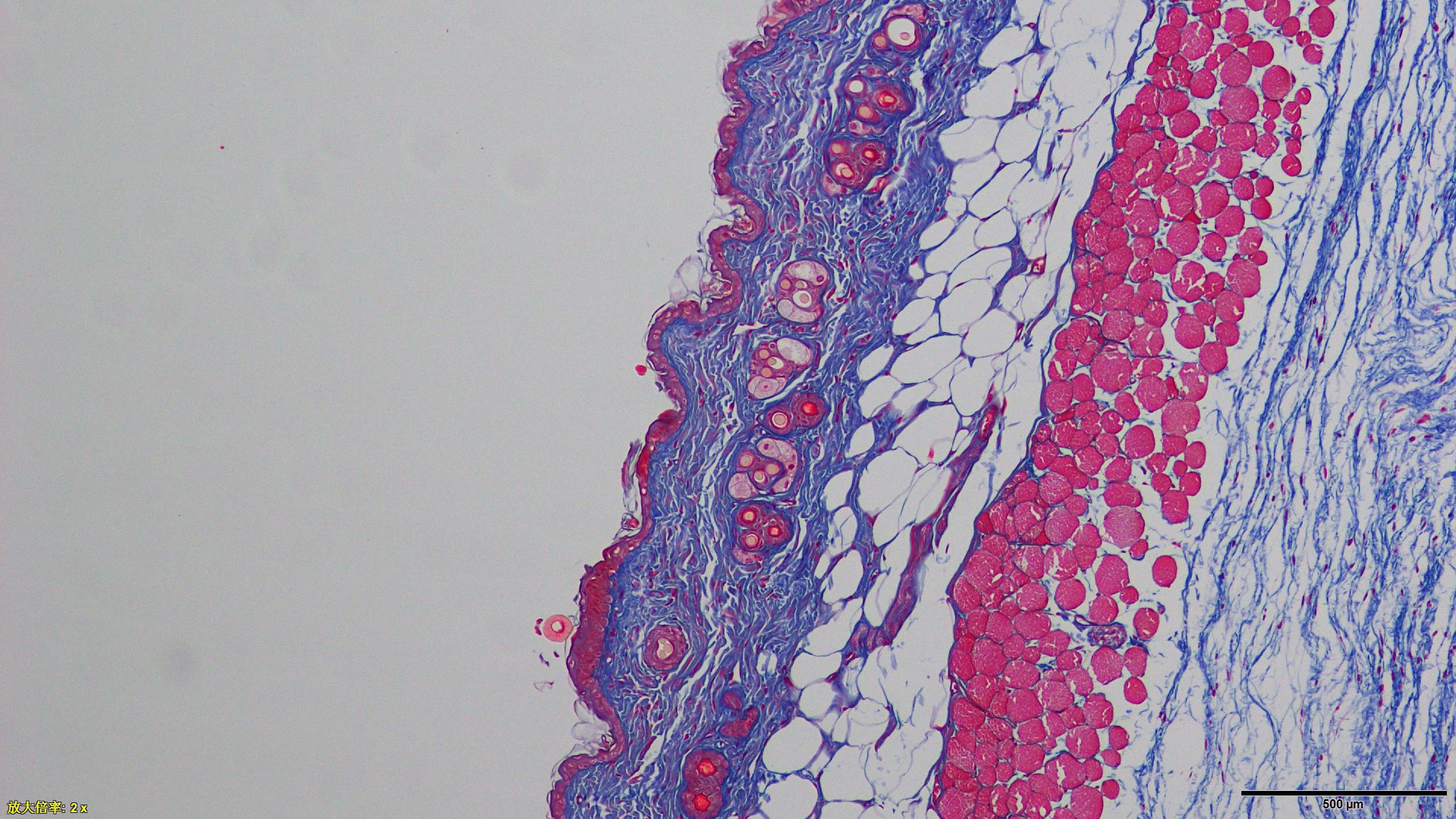

Supplement: Supplementary file 2 [file Data_Sheet_1.ZIP › SECTION/SKIN/Masson/CQPC04.jpg]

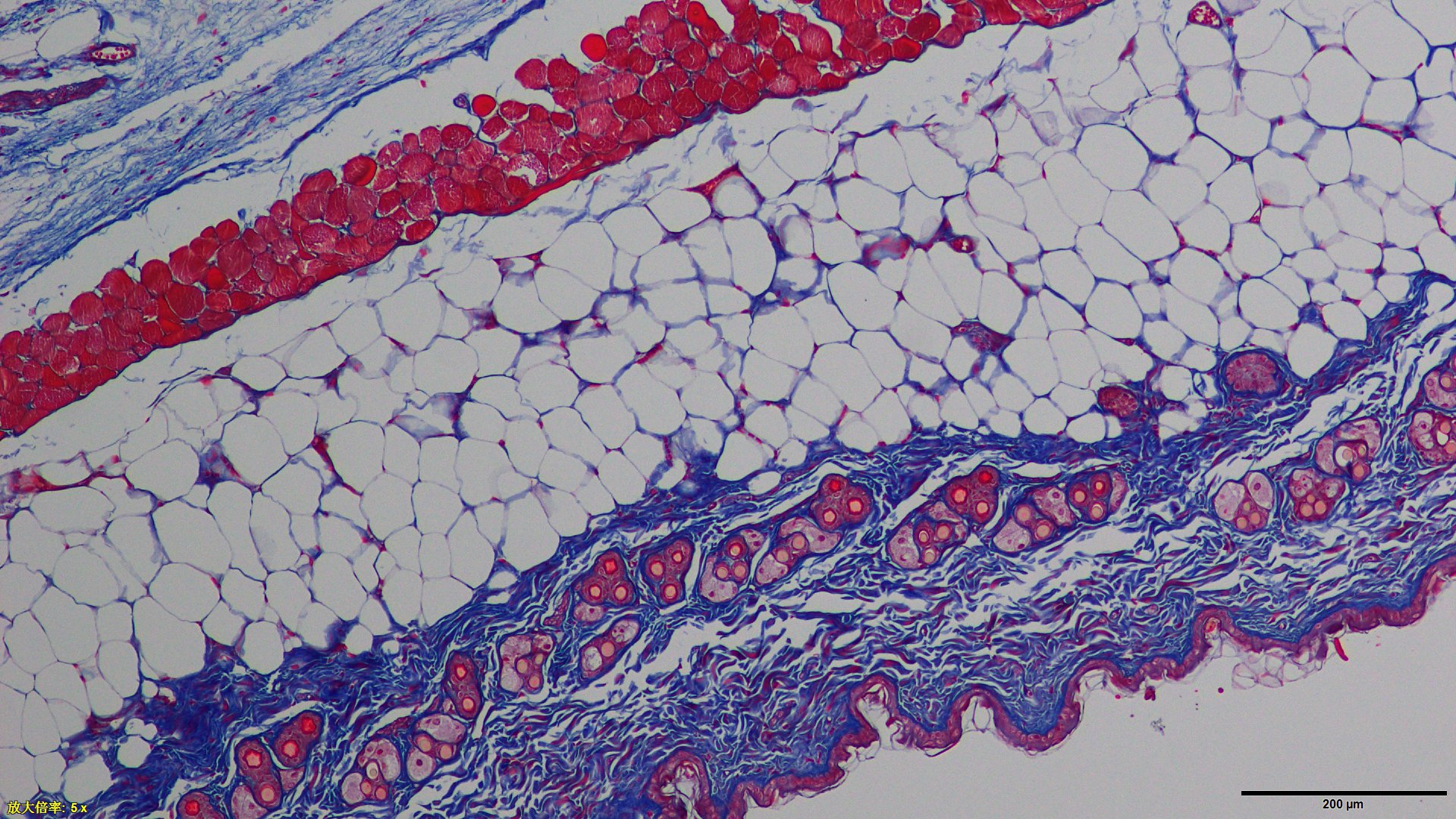

Supplement: Supplementary file 2 [file Data_Sheet_1.ZIP › SECTION/SKIN/Masson/NO.jpg]

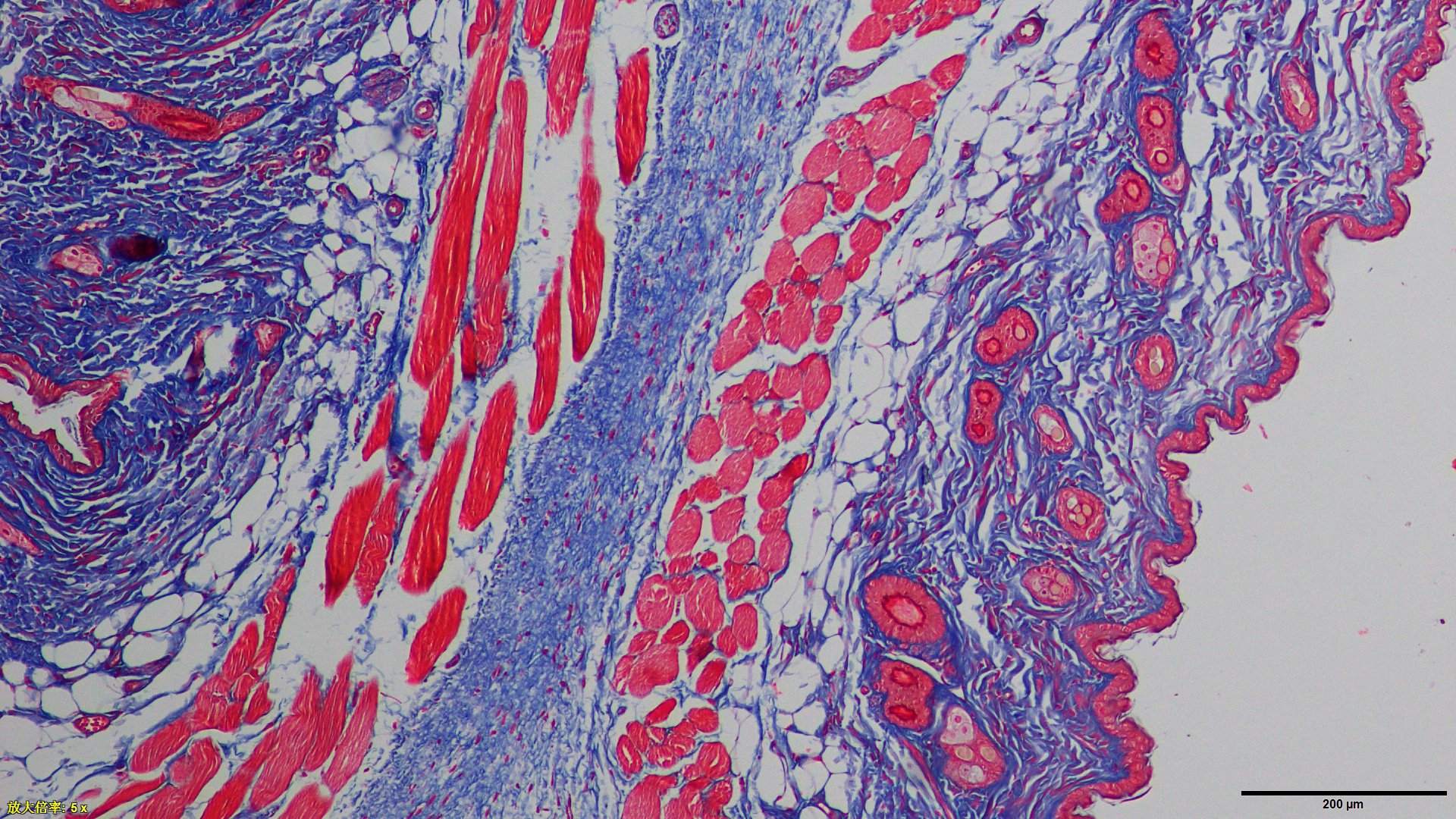

Supplement: Supplementary file 2 [file Data_Sheet_1.ZIP › SECTION/SKIN/Masson/VC.jpg]

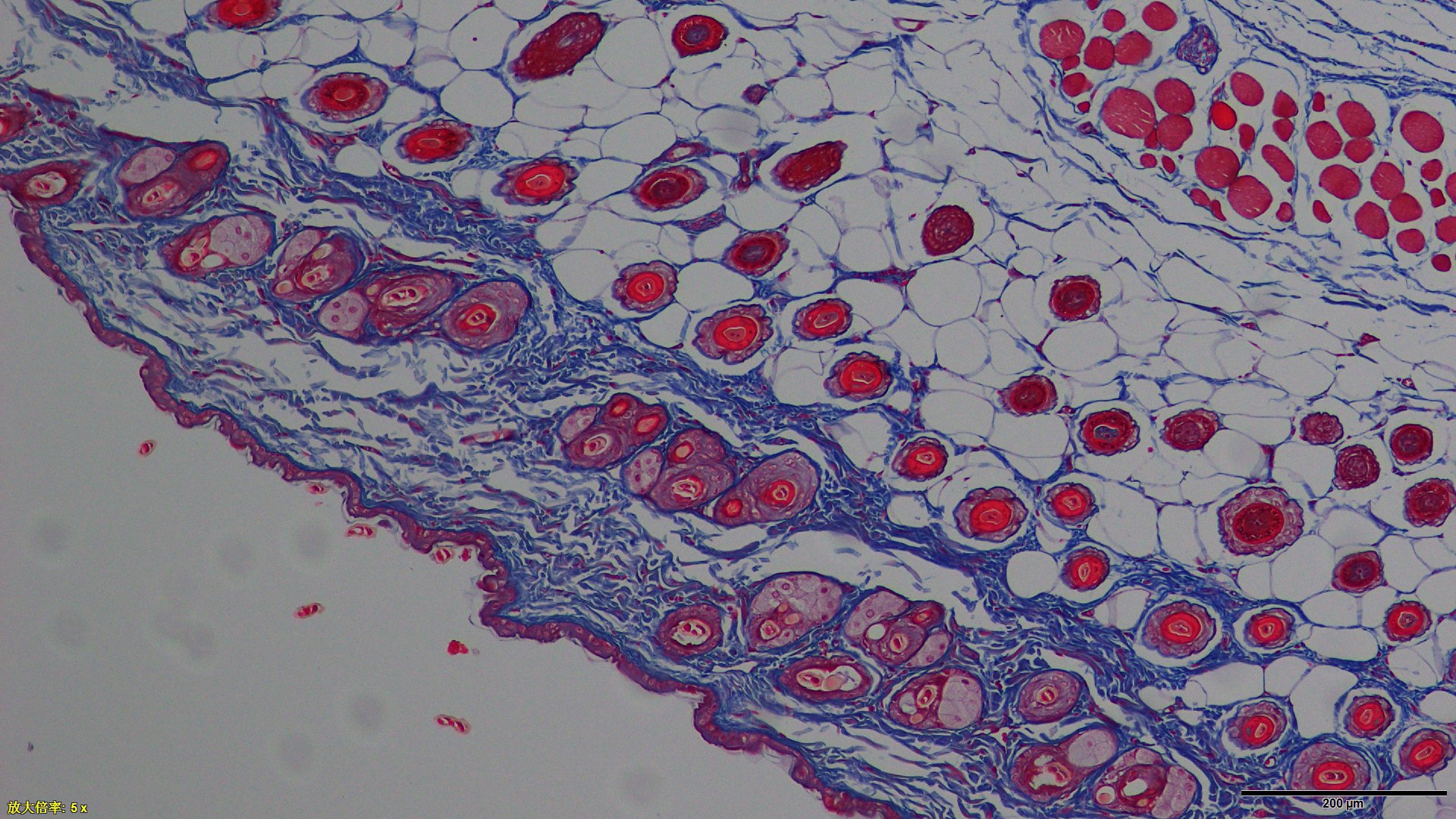

Supplement: Supplementary file 2 [file Data_Sheet_1.ZIP › SECTION/SKIN/Masson/模型.jpg]

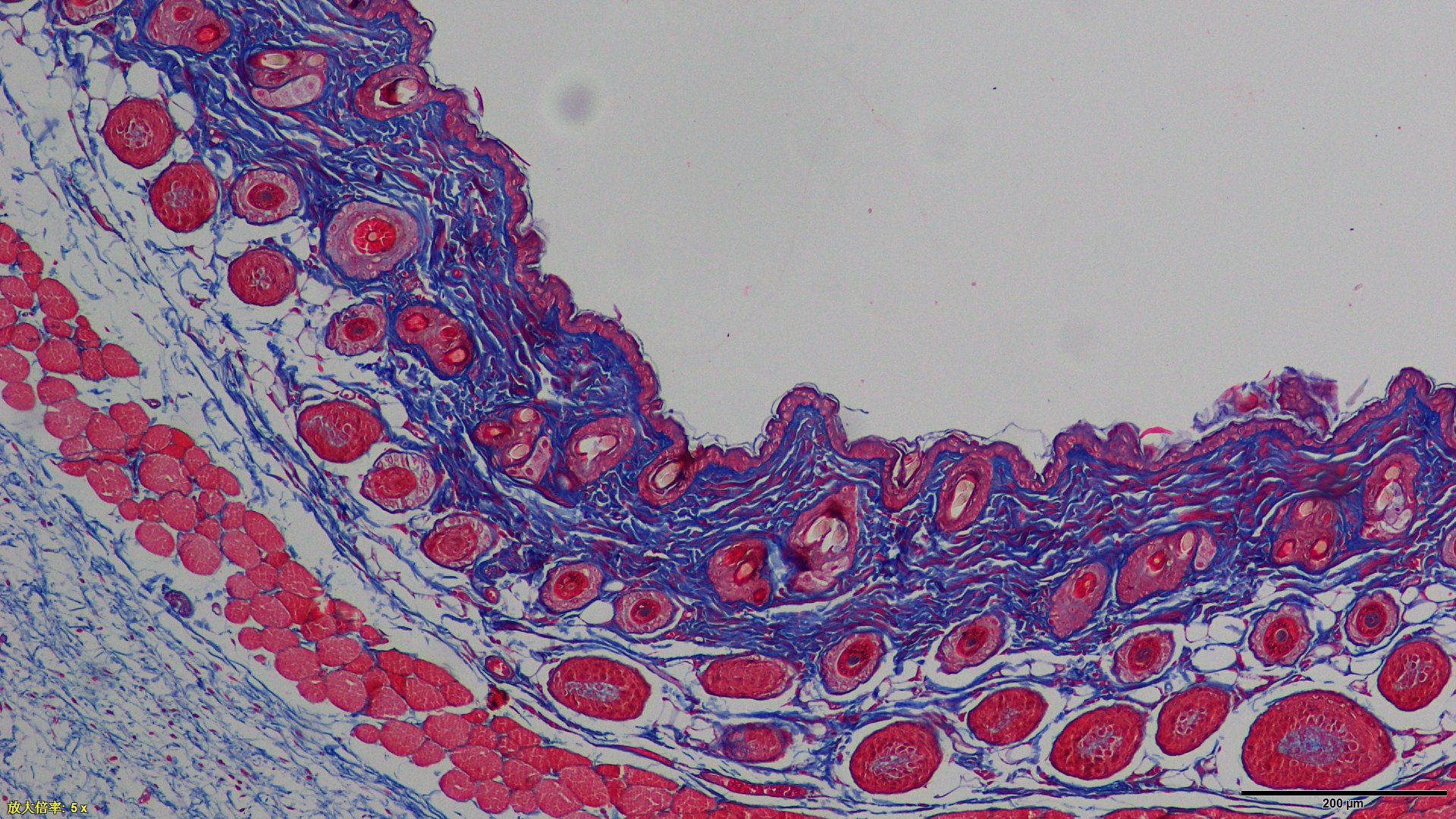

Supplement: Supplementary file 2 [file Data_Sheet_1.ZIP › SECTION/SKIN/Masson/正常.jpg]

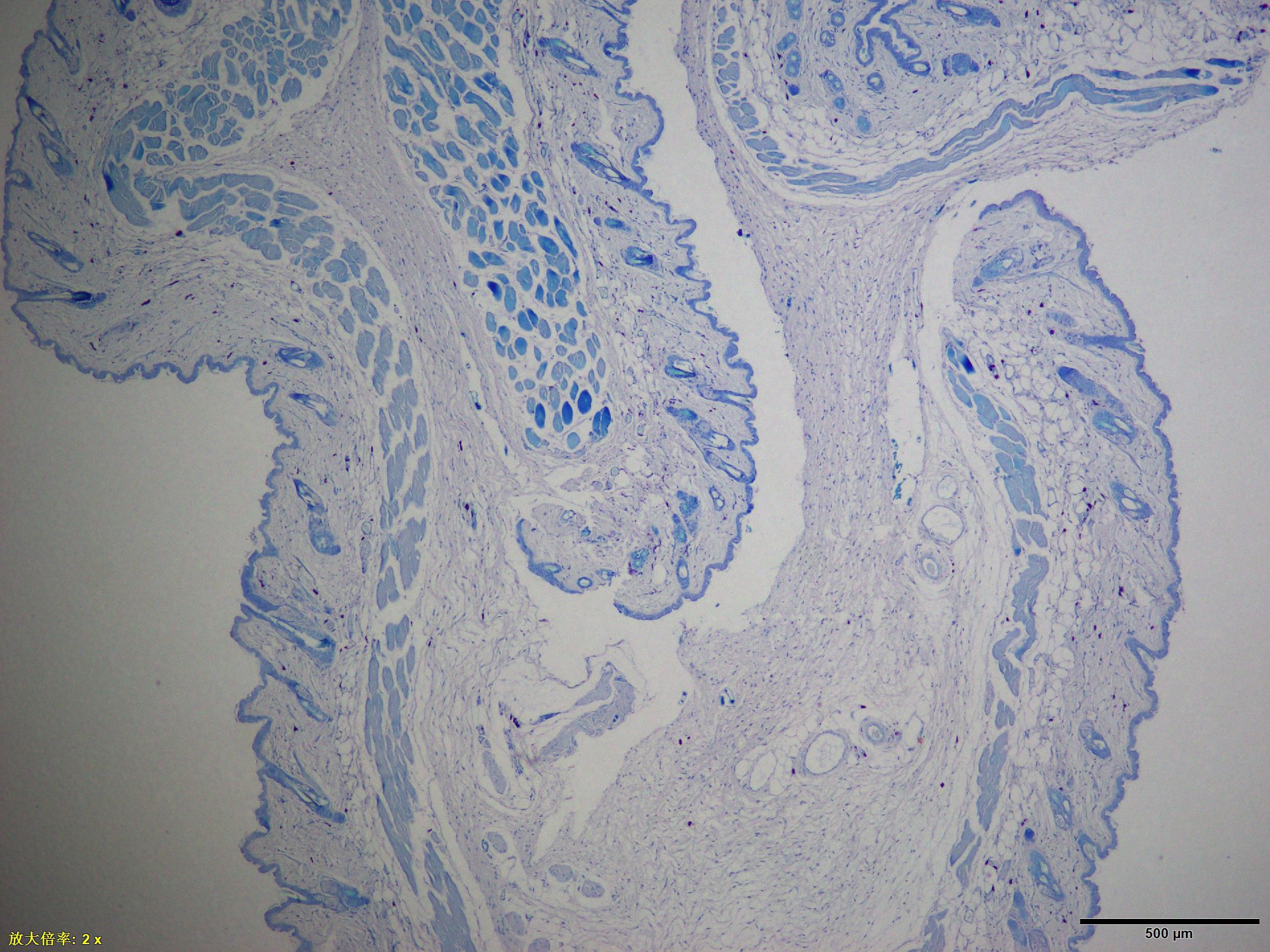

Supplement: Supplementary file 2 [file Data_Sheet_1.ZIP › SECTION/SKIN/TB/CQPC04.jpg]

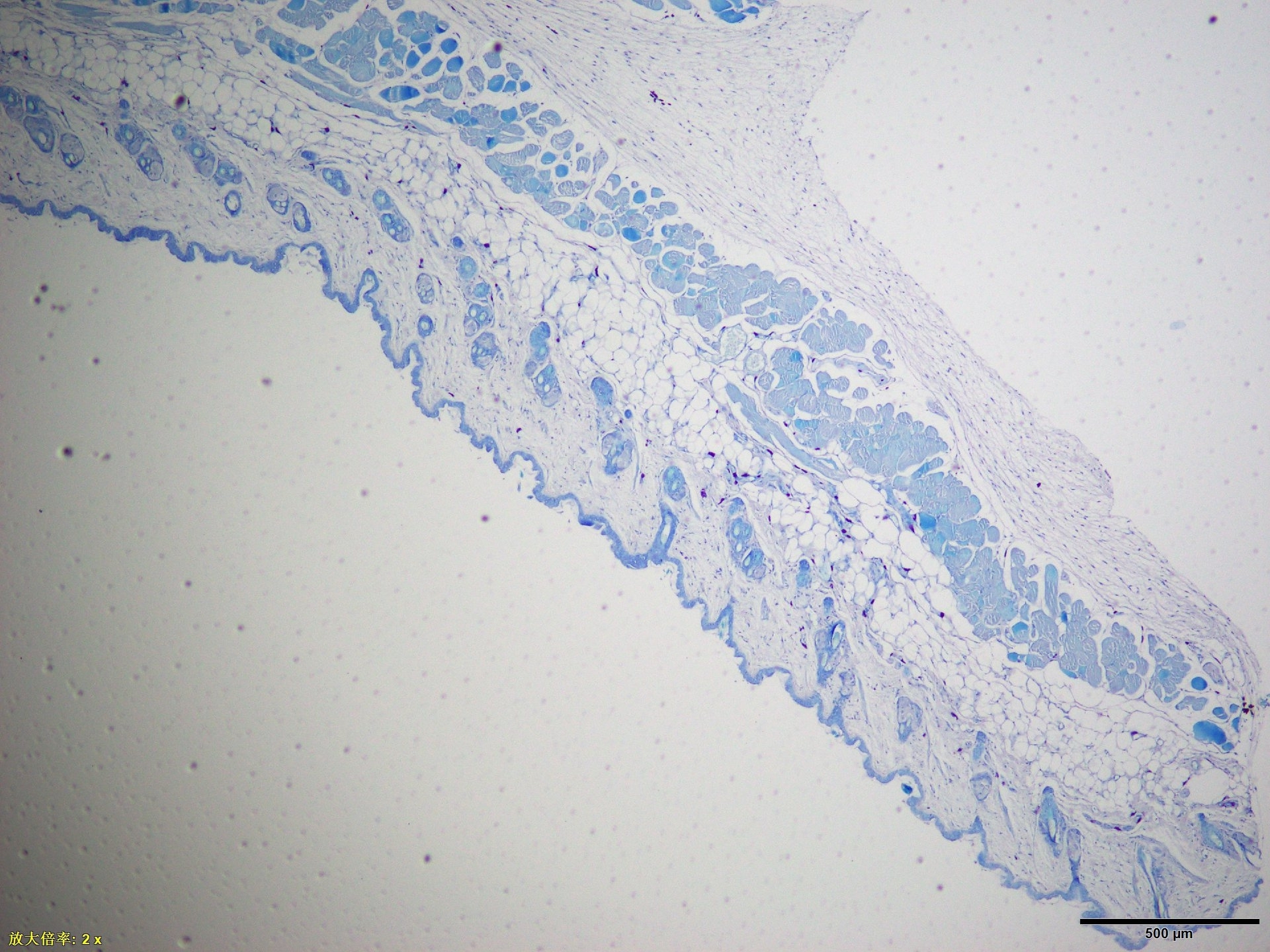

Supplement: Supplementary file 2 [file Data_Sheet_1.ZIP › SECTION/SKIN/TB/NO.jpg]

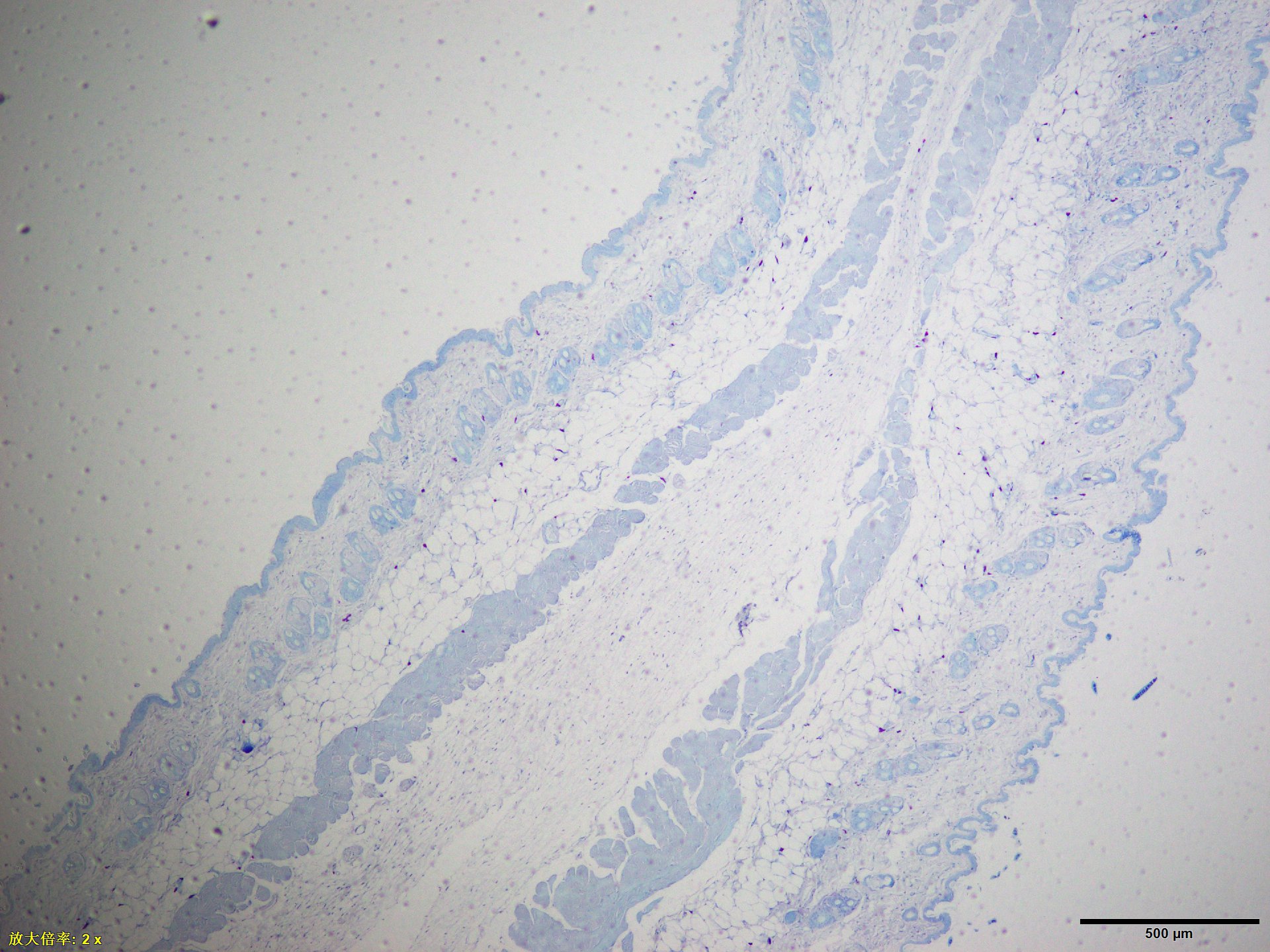

Supplement: Supplementary file 2 [file Data_Sheet_1.ZIP › SECTION/SKIN/TB/VC.jpg]

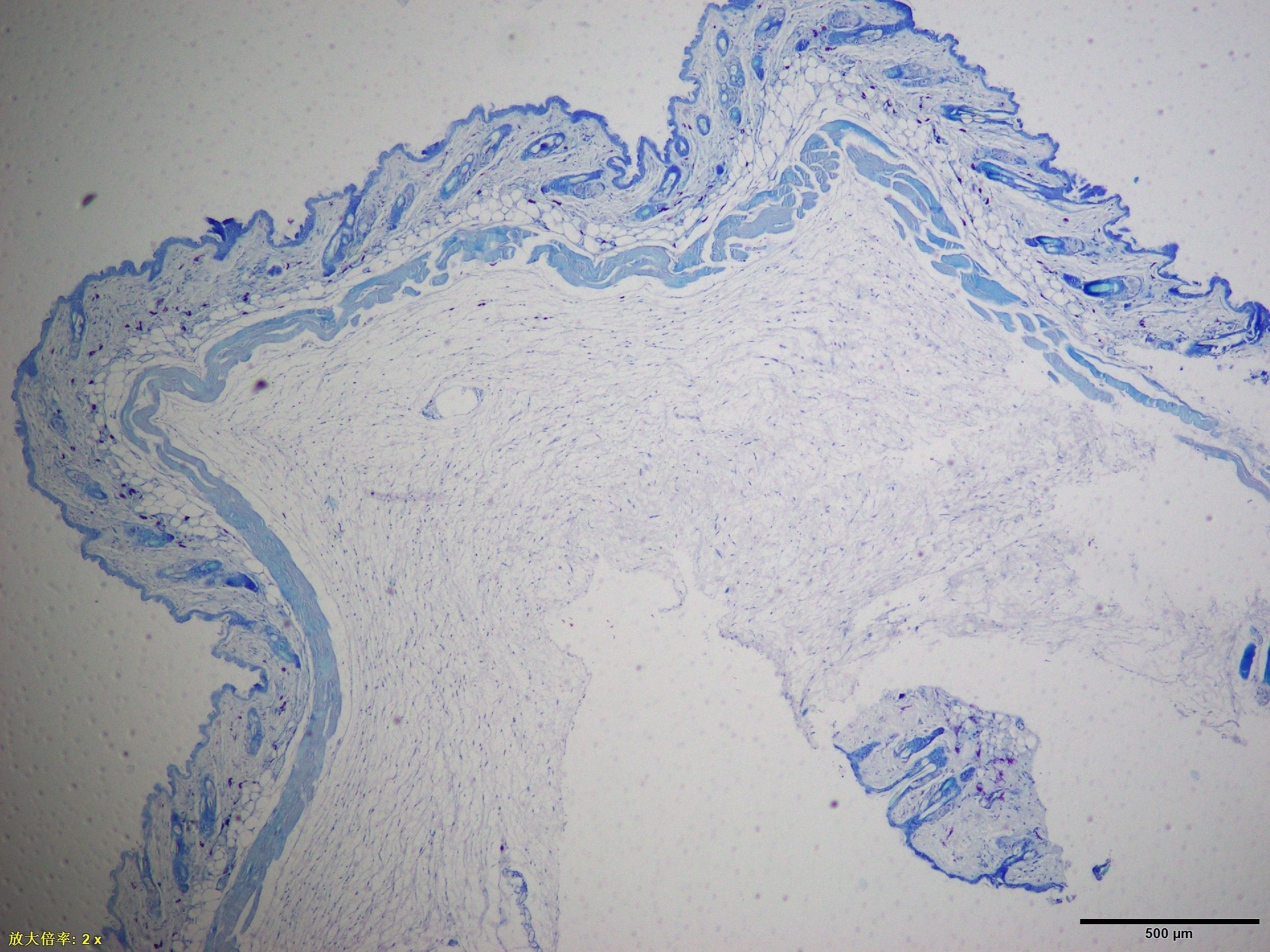

Supplement: Supplementary file 2 [file Data_Sheet_1.ZIP › SECTION/SKIN/TB/模型.jpg]

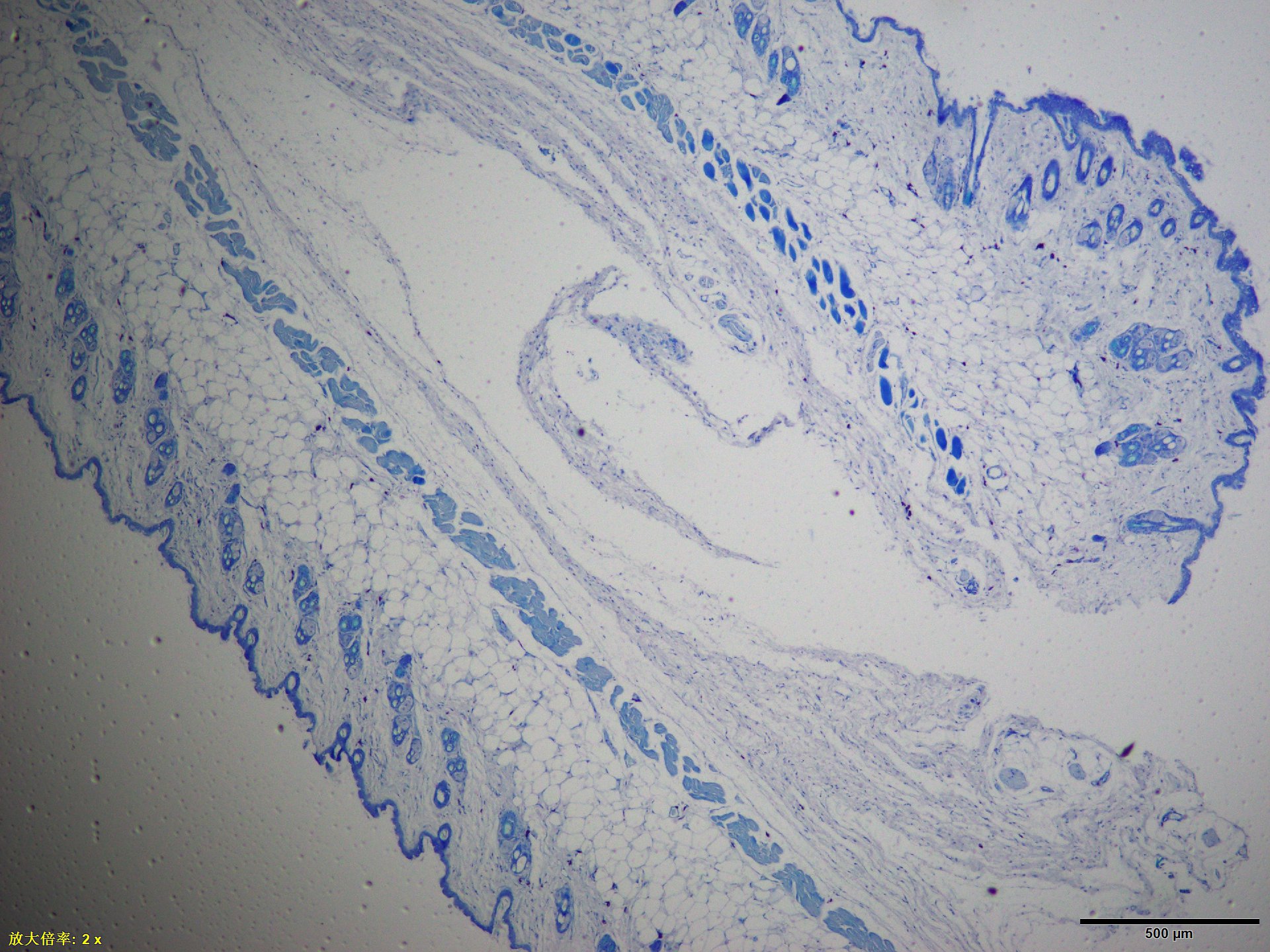

Supplement: Supplementary file 2 [file Data_Sheet_1.ZIP › SECTION/SKIN/TB/正常.jpg]

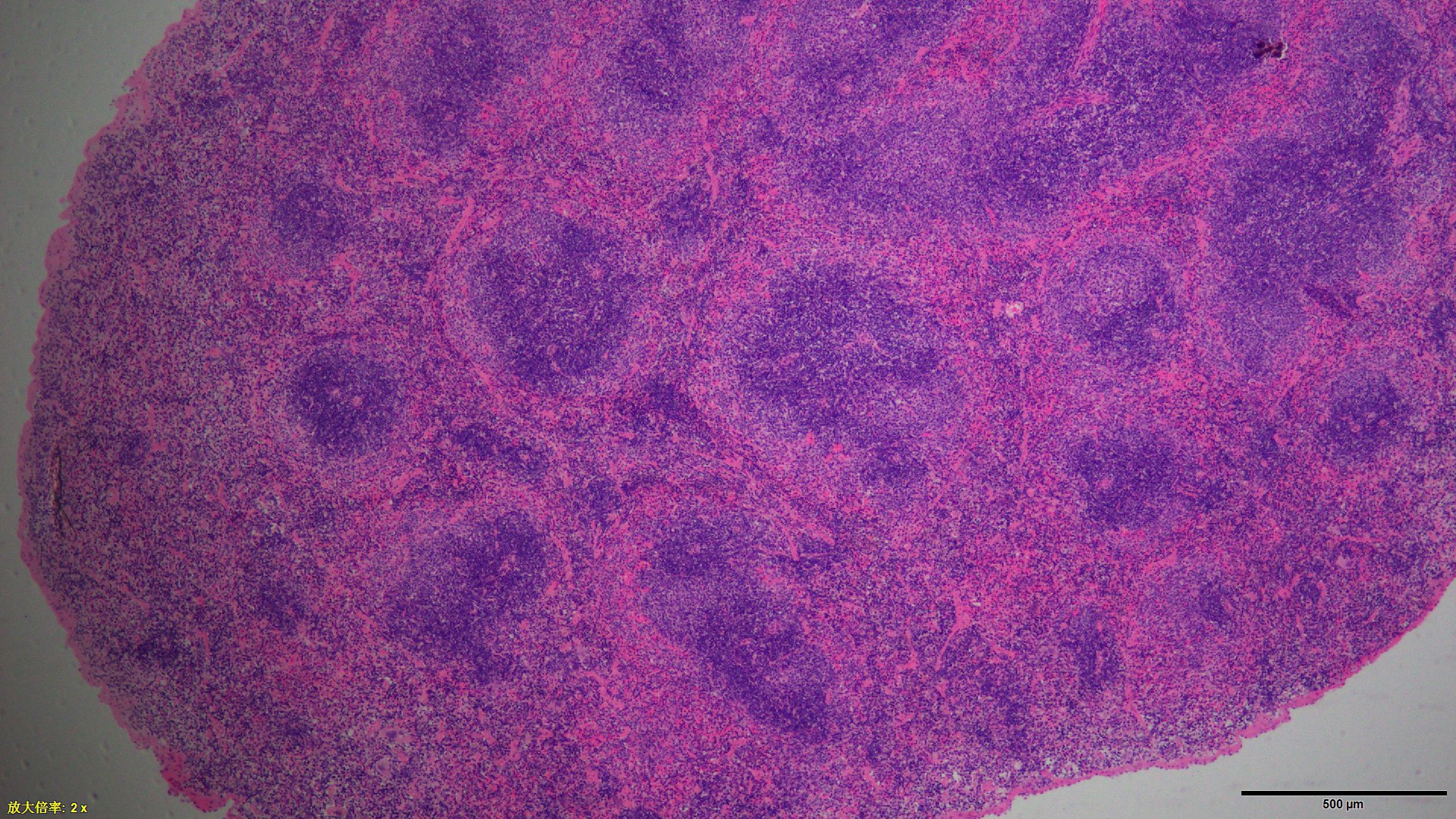

Supplement: Supplementary file 2 [file Data_Sheet_1.ZIP › SECTION/SPLEEN/04 11-4.jpg]

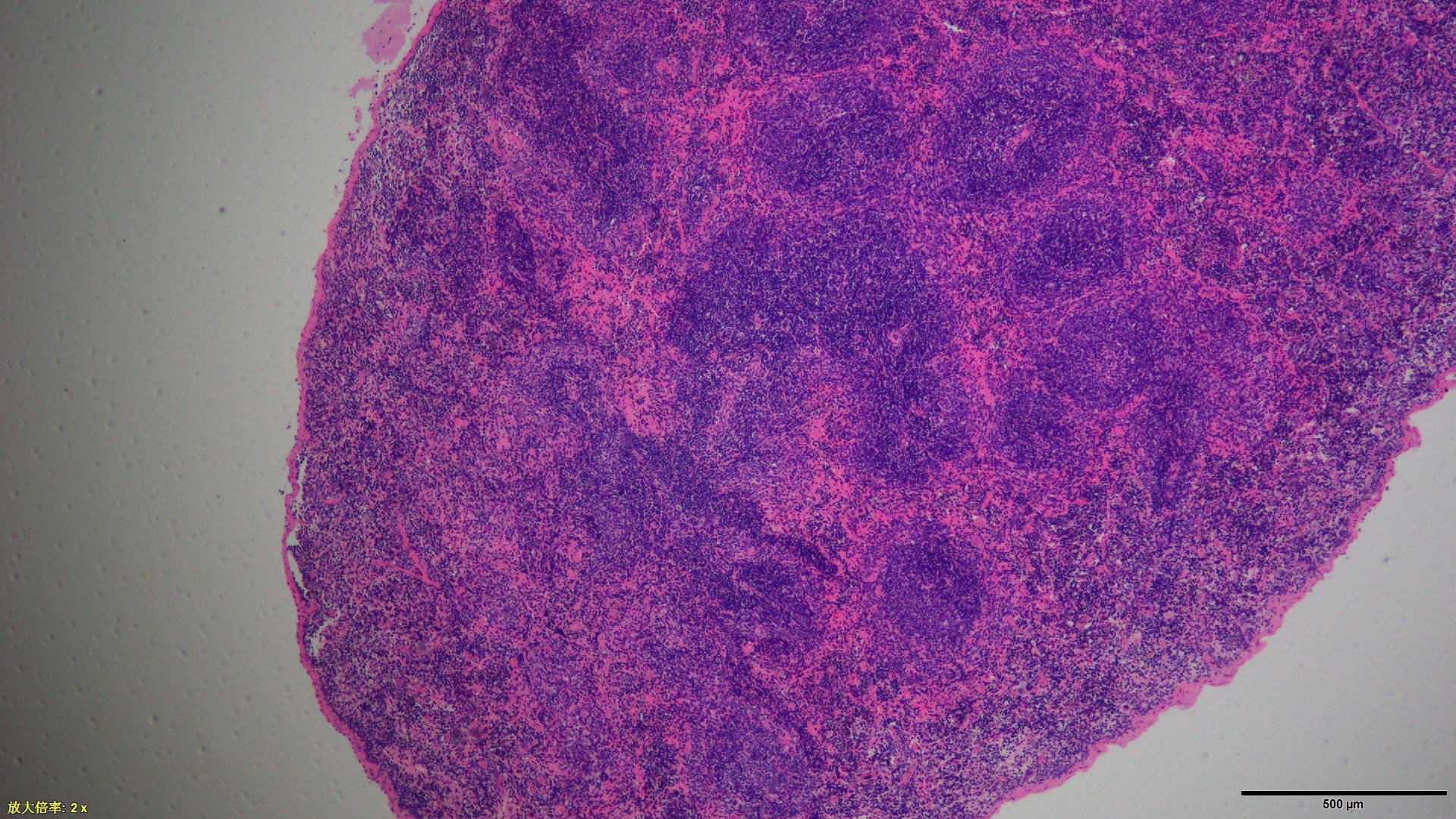

Supplement: Supplementary file 2 [file Data_Sheet_1.ZIP › SECTION/SPLEEN/NO3-1.jpg]

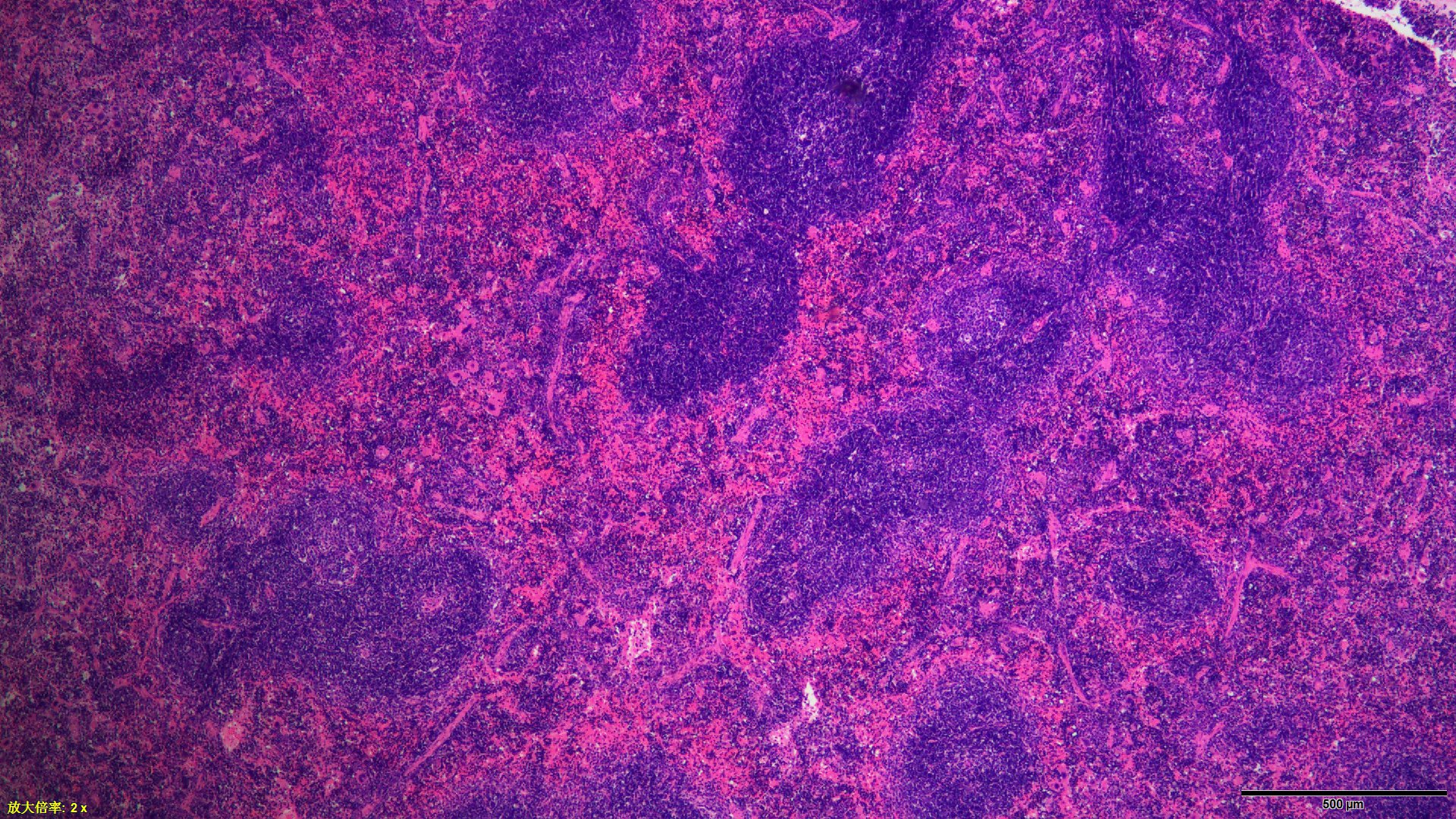

Supplement: Supplementary file 2 [file Data_Sheet_1.ZIP › SECTION/SPLEEN/VC6-3.jpg]

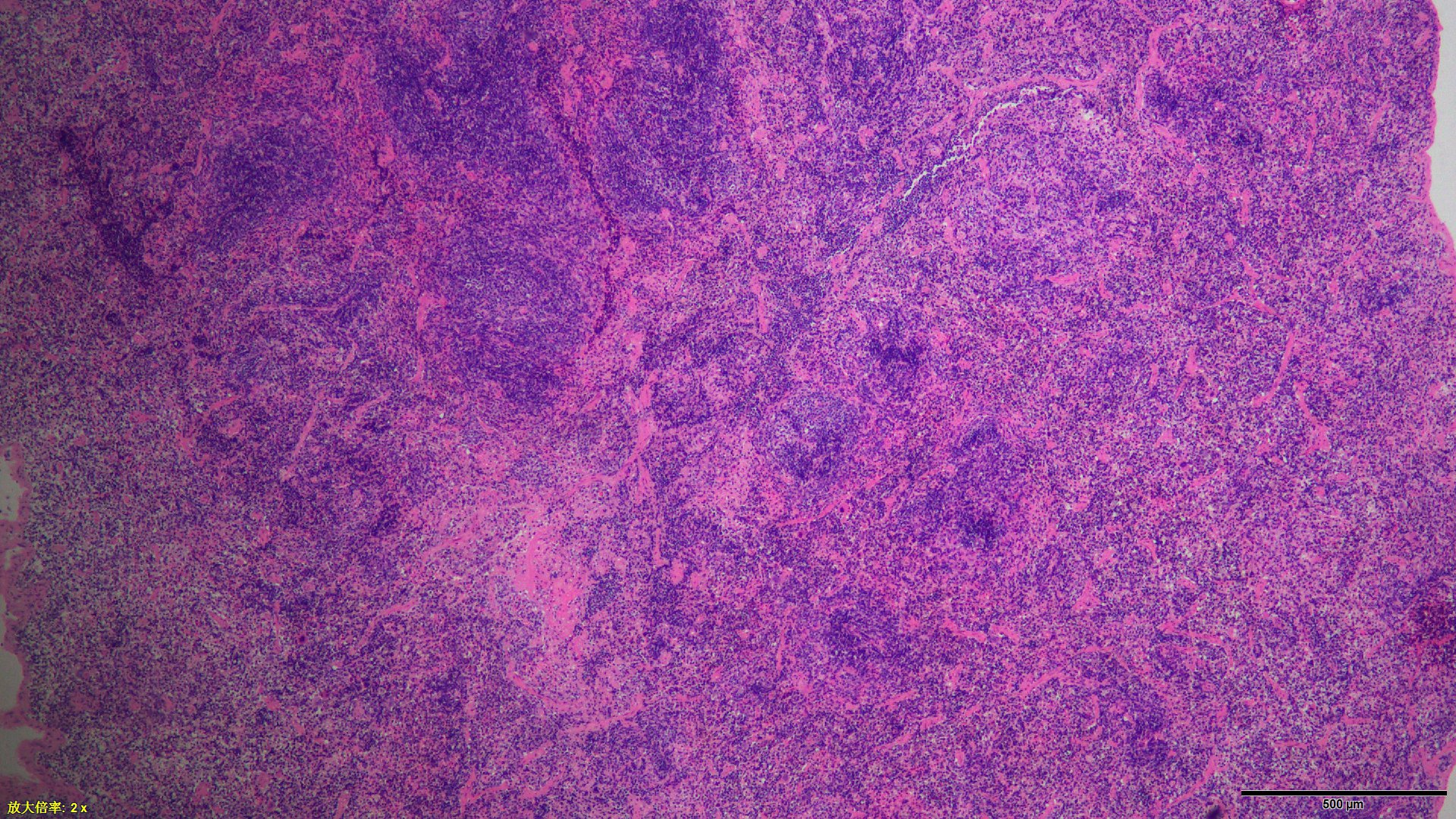

Supplement: Supplementary file 2 [file Data_Sheet_1.ZIP › SECTION/SPLEEN/模型6-3.jpg]

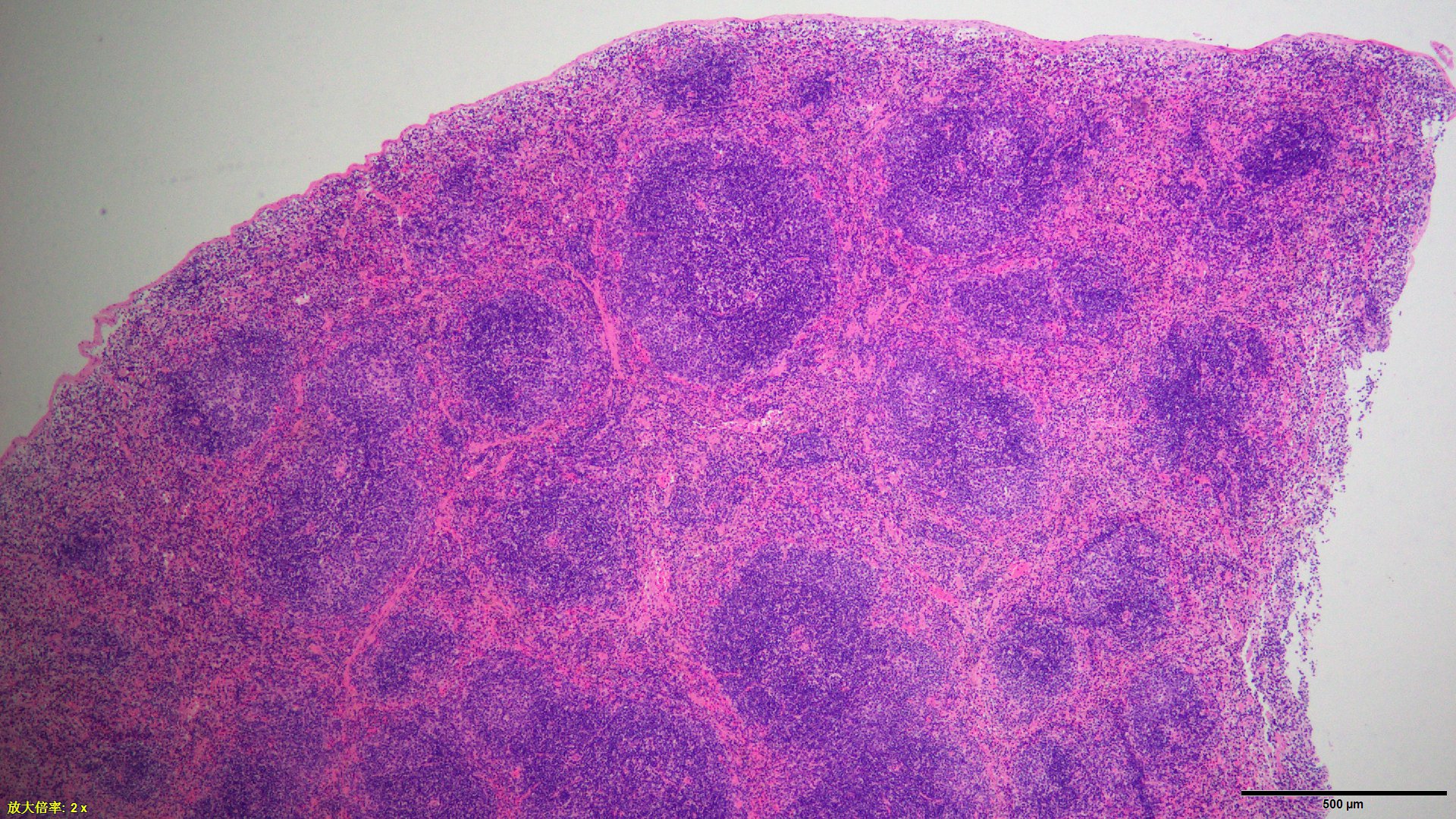

Supplement: Supplementary file 2 [file Data_Sheet_1.ZIP › SECTION/SPLEEN/正1-2.jpg]
